# Maternal high-fat diet modifies myelin organization, microglial interactions, and results in social memory and sensorimotor gating deficits in adolescent mouse offspring

**DOI:** 10.1016/j.bbih.2021.100281

**Published:** 2021-06-09

**Authors:** Maude Bordeleau, Lourdes Fernández de Cossío, Chloé Lacabanne, Julie C. Savage, Nathalie Vernoux, Mallar Chakravarty, Marie-Ève Tremblay

**Affiliations:** a Integrated Program in Neuroscience, McGill University, Montréal, QC, Canada; b Axe Neurosciences, Centre de Recherche du CHU de Québec – Université Laval, Québec, QC, Canada; c Department of Neurosciences, University of California, La Jolla, San Diego, CA, USA; d Cerebral Imaging Center, Douglas Mental Health University Institute, McGill University, Montréal, QC, Canada; e Department of Psychiatry, McGill University, Montréal, QC, Canada; f Department of Biological and Biomedical Engineering, McGill University, Montréal, QC, Canada; g Département de Médecine Moléculaire, Université Laval, Québec, QC, Canada; h Department of Neurology and Neurosurgery, McGill University, Montréal, QC, Canada; i Division of Medical Sciences, University of Victoria, Victoria, BC, Canada; j Department of Biochemistry and Molecular Biology, Faculty of Medicine, The University of British Colombia, Vancouver, BC, Canada

**Keywords:** Maternal high-fat diet, Myelin, Cytosolic channels, Oligodendrocytes, Microglia, Adolescence, Mouse

## Abstract

Prenatal exposure to maternal high-fat diet (mHFD) acts as a risk factor for various neurodevelopmental alterations in the progeny. Recent studies in mice revealed that mHFD results in both neuroinflammation and hypomyelination in the exposed offspring. Microglia, the brain-resident macrophages, play crucial roles during brain development, notably by modulating oligodendrocyte populations and performing phagocytosis of myelin sheaths. Previously, we reported that mHFD modifies microglial phenotype (i.e., morphology, interactions with their microenvironment, transcripts) in the hippocampus of male and female offspring. In the current study, we further explored whether mHFD may induce myelination changes among the hippocampal-corpus callosum-prefrontal cortex pathway, and result in behavioral outcomes in adolescent offspring of the two sexes. To this end, female mice were fed with control chow or HFD for 4 weeks before mating, during gestation, and until weaning of their litter. Histological and ultrastructural analyses revealed an increased density of myelin associated with a reduced area of cytosolic myelin channels in the corpus callosum of mHFD-exposed male compared to female offspring. Transcripts of myelination-associated genes including *Igf1* –a growth factor released by microglia– were also lower, specifically in the hippocampus (without changes in the prefrontal cortex) of adolescent male mouse offspring. These changes in myelin were not related to an altered density, distribution, or maturation of oligodendrocytes, instead we found that microglia within the corpus callosum of mHFD-exposed offspring showed reduced numbers of mature lysosomes and increased synaptic contacts, suggesting microglial implication in the modified myelination. At the behavioral level, both male and female mHFD-exposed adolescent offspring presented loss of social memory and sensorimotor gating deficits. These results together highlight the importance of studying oligodendrocyte-microglia crosstalk and its involvement in the long-term brain alterations that result from prenatal mHFD in offspring across sexes.

## Highlights

• mHFD induces myelination changes specifically in male adolescent offspring.
• mHFD increases microglia-synapse contacts in adolescent offspring of both sexes.
• mHFD reduces microglial mature lysosomes in adolescent offspring of both sexes.
• mHFD impairs social novelty and sensorimotor gating during adolescence.

## 1 Introduction

The brain is 60% lipids, making it the most lipid-rich organ in the body (O'Brien and Sampson, 1965). The main lipid component of the brain is myelin, produced by oligodendrocytes, and which insulates axons for efficient neuronal signal transmission (Domingues et al., 2018). Myelination, the ensheathment of axons with myelin, begins in the third trimester of pregnancy and continues beyond adolescence in human (Knuesel et al., 2014). This process requires dynamic interactions between oligodendrocytes and cues in their microenvironment, including the extracellular matrix (Domingues et al., 2018), and other types of glial cells (Domingues et al., 2016). During development, both astrocytes and microglia intervene in oligodendrocyte differentiation and survival, and they are implicated in physiological demyelination and remyelination processes later in life (Domingues et al., 2016). Myelin anomalies associated with myelinogenic gene expression, hypomyelination, and a disruption of oligodendrocyte maturation, impair neuronal communication and are observed in several neurodevelopmental disorders (Fatemi et al., 2009; Dries et al., 2016; Olmos-Serrano et al., 2016; Makinodan et al., 2017; Stassart et al., 2018). These anomalies can emerge after exposure to environmental risk factors, such as an inadequate or imbalanced dietary intake of fats, especially during the critical period of prenatal development (Fatemi et al., 2009; Graf et al., 2016; Teissier et al., 2020; Bordeleau et al., 2021).

During pregnancy, the excess intake of dietary fats (saturated or unsaturated) has been shown to disrupt myelination differently in male *vs* female pubertal offspring (Graf et al., 2016). Using a maternal high-fat diet (mHFD) mouse model, Graf and colleagues revealed, only in male offspring, a reduced number of oligodendrocytes in the lateral cortex at postnatal day (P) 7 and a reduced myelin area in the medial cortex at P21 (Graf et al., 2016). Concomitant to these changes, plasma circulating cytokines were increased in HFD-fed dams [interleukin (IL)-2, IL-6, IL-4] and their offspring (P7 male and P21 female: IL-5). Whole brain homogenates also had altered neuroinflammatory genes (P0 male: *Nurr1*; P21 male: *Il-1b*, *Nurr77*; P21 female: *Nurr77*) in the mHFD-exposed offspring (Graf et al., 2016). Nevertheless, it remains unclear how the inflammatory and myelination changes are linked together.

In the developing brain, immune cells and their mediators are critical for proper neurodevelopmental processes (Bordeleau et al., 2019, Morimoto and Nakajima, 2019). In mice, Iba1-positive (^+^) microglia that originate from the embryonic yolk sac transiently accumulate after birth in white matter regions including the corpus callosum (Hagemeyer et al., 2017). Similarly, neonatal microglial subpopulations in mice, CD11c^+^ (Wlodarczyk et al., 2017) and GPNMB^+^/CLEC7A^+^ (Li et al., 2019), were reported to partake in postnatal myelination by contributing to oligodendrocyte maturation and myelin formation (Wlodarczyk et al., 2017; Li et al., 2019), as well as phagocytic elimination of dying oligodendrocytes (Li et al., 2019; Nemes-Baran et al., 2020) and ectopic myelin sheaths (Hughes and Appel, 2020). In mice, active myelination of the brain peaks around P21 until P30, which also corresponds to the beginning of a myelin maintenance phase that continues beyond adulthood (Flores et al., 2008).

These findings indicate that mHFD-induced inflammation may affect myelination directly by altering oligodendrocytes or indirectly via microglial functional interactions with myelin and oligodendrocytes. To test these two hypotheses, we used a mHFD (4 weeks prior to pregnancy, throughout gestation, and during nurturing) mouse model that we have characterized recently (Bordeleau et al., 2020). In the current study, by combining molecular, cellular and ultrastructural cytochemical techniques, we investigated changes in myelin organization, oligodendrocyte population and the functional interactions of microglia with this process, in the two sexes, at P30 corresponding to early adolescence. Our work sheds light on the effects of mHFD on myelin organization (optical density, cytosolic myelin channels) within the corpus callosum –the main collection of myelinated axons of the brain, the consequences on molecular meditators underlying the myelination process in two regions receiving projections from the corpus callosum (hippocampus, prefrontal cortex) of male and female adolescent offspring, as well as on adolescent behaviors associated with neurodevelopmental disorders (Leonardo and Hen, 2008; Mena et al., 2016; Isaksson et al., 2019).

## 2 Materials and methods

### 2.1 Animal

Animal protocol was approved by McGill University Animal Care Committee under the guidelines of the Canadian Council on Animal Care. Animals were submitted to standard housing condition: 12h dark/light cycle (08:00–20:00) with free access to water and food. C57BL/6N female mice aged 5–6 weeks were obtained from Charles River and habituated to pair-housing for one week. At 6–7 weeks, the mice were randomly assigned to be fed with either control diet chow (CD; Teklad, 2014; ENVIGO, Indianapolis, IN, United States) or HFD (diet rich in saturated/unsaturated fat; 60% kcal by lipids that results in a similar amount of calories; Teklad TD.06414, ENVIGO) *ad libitum* for 4 weeks prior to mating, then throughout gestation and nurturing, until weaning of their litter, which led to greater fat deposition in the dams as previously described by our team (Bordeleau et al., 2020). After mating, pregnant dams were housed single or alone with their litter. At P21, offspring were weaned, split by sex, 2–5 per cage, and fed with CD. Animals with major anomalies (unopened or abnormal eyes, dwarfs or severe tooth malformation) were excluded from subsequent protocol. Of note, the occurrence of these anomalies did not differ between diet groups. For each experiment, one or two offspring were used per litter to prevent litter-driven effect. A total of three cohorts of animals randomized by litter were used either for tissue collection at P30 or behavioral characterization between P32 and P40.

### 2.2 Histology analysis

For histological analysis, anesthetized mice (n ​= ​5 animals/diet/sex) were transcardially flushed with ~15 ​mL of [50 ​mM] phosphate-buffered saline (PBS; pH ​= ​7.4) and perfused with ~180 ​mL 4% paraformaldehyde (PFA) diluted in [100 ​mM] phosphate buffer (PB; pH ​= ​7.4). Brains were post-fixed 24h in 4% PFA then switched to 30% glucose solution (diluted in [50 ​mM] PBS, pH ​= ​7.4; Sigma) for 48h before being flash-frozen for storage at −80 ​°C until cutting. Frozen fixed brains were then cut into 30 ​μm-coronal sections using a CM3050S cryostat (Leica Biosystem, Wetzlar, Germany) and stored freely floating in cryoprotectant solution (30% ethylene glycol, 30% glycerol in PBS) at −20 ​°C until use.

#### 2.2.1 Myelin of the corpus callosum

Sequential coronal sections containing the corpus callosum across the mouse brain (every ~200 ​μm, ~8–9 sections/brain, n ​= ​5 animals/diet/sex) were mounted on positively charged slides and let dry overnight. Brain-mounted slides were rinsed in [50 ​mM] tris-buffered saline (TBS) for 5min, then incubated in 1% Sudan Black B (cat# 3545, MilliporeSigma, Burlington, MA, United States) in ethylene glycol for 30min at room temperature (RT), and rinsed 2× 5min in TBS before being coverslipped in aqueous mounting medium (SouthernBiotech, Birmingham, AB, United States).

Sections containing the corpus callosum were imaged in a single plane at 10X using an Axio Imager M2 epifluorescence microscope equipped with an AxioCam ICC 3 camera (Zeiss, Oberkochen, Germany). Individual regions of the corpus callosum (rostrum, genu, body and splenium) were double-blindly analyzed, separately as they contain connections between different brain regions (Goldstein et al., 2020). Using ImageJ (v1.51j8; National Institute of Health, Bethesda, MD, United States), corpus callosum area was traced using the “Freehand” tool, and area occupied as well as mean grey value of the corpus callosum were automatically measured for each section using the following formula, where minimal intensity value is black (0) and maximum intensity value is white (255):

$$Calibrated\;\text{OD}=\log{\;}_{10}\left(\frac{I_{min}}{I_{max}}\right)=\log{\;}_{10}\left(\frac{Mean\;gray\;value_{background}}{Mean\;gray\;value_{corpus\;callosum}}\right)$$

#### 2.2.2 Oligodendrocyte maturation

Three to four brain sections per animal containing the rostrum of the corpus callosum (Bregma 1.69–1.33 ​mm, stereotaxic atlas of Paxinos and Franklin 4^th^ edition (Paxinos and Franklin, 2013); n ​= ​5 animals/diet/sex) were selected and washed 5× 5min in PBS. Freely floating sections were then incubated in 0.1% NaBH_4_ in [50 ​mM] PBS (pH ​= ​7.4) for 30min to quench tissue autofluorescence. Sections were washed 5× 5min in PBS, then blocked for 30min in blocking buffer (BB; 5% donkey serum, 0.5% gelatin, 0.3% Triton X-100 in [50 ​mM] PBS, pH ​= ​7.4). After blocking, brains sections were incubated overnight at 4 ​°C with a cocktail of primary antibodies: rabbit anti-Nogo-A (1:600 in BB; produced and graciously given by Dr. Vincent Pernet) and goat anti-Olig2 (1:400 in BB; cat# AF2418, R&D Systems, Minneapolis, MN, United States). The following day, tissue sections were washed 5× 5min in PBS, then incubated with secondary antibodies: donkey anti-rabbit Alexa Fluor 647-conjugated (1:500 in PBS; cat# A31573, Invitrogen, Thermo Fisher Scientific, Waltham, MA, United States) and donkey anti-goat Alexa Fluor 568-conjugated (1:500 in PBS; cat# A11057, Invitrogen, Thermo Fisher Scientific) for 2h at RT. Sections were rinsed 5× 5min in PBS, incubated with DAPI ([0.2 ​ng/mL] in PBS; cat# D1306, Invitrogen) for 5min, and rinsed 5× 5min in [100 ​mM] PB (pH ​= ​7.4) before mounting on slides. Mounted slides were let to dry overnight and coverslipped in aqueous Fluoromount G mounting medium (SouthernBiotech).

Six to eight rostra of the corpus callosum per animal were imaged in a single plane at 20X using an Axio Imager M2 epifluorescence microscope equipped with an AxioCamMRm (Zeiss). The analyses were performed in double-blind using ImageJ. Rostra were traced using the “Freehand” tool to determine their area. Cell counts of Nogo-A^+^/Olig2^+^ cells (mature oligodendrocytes) (Kuhlmann et al., 2007) and Nogo-A-negative (^-^)/Olig2^+^ cells (oligodendrocyte progenitors and immature oligodendrocytes) were performed to measure cell density of the two populations in a semi-automatic manner using the “Convert Mask” and “Analyze Particles” plugins. Olig2 and Nogo-A “Integrated Density” measurement was used to quantify fluorescence intensity.

#### 2.2.3 Microglial number, distribution, and morphology

Three to four brain sections per animal containing the rostrum of the corpus callosum (Bregma 1.42 to 1.24, stereotaxic atlas of Paxinos and Franklin 4^th^ edition (Paxinos and Franklin, 2013); n ​= ​4–5 animals/diet/sex) were selected and washed 5× 5min in PBS. Then, tissues were incubated for 40min in 0.1% NaBH_4_ in PBS, washed 5× 5min in PBS, and incubated in BB (5% donkey serum, 0.5% gelatin, 0.1% Triton X-100 in TBS). After blocking, sections were incubated overnight at 4 ​°C with a cocktail of primary antibodies: mouse anti-Iba1 (1:190 in BB; cat# MABN92, MilliporeSigma) and rabbit anti-TMEM119 (1:300 in BB; cat# ab209064, Abcam). The next day, tissues were washed 5× 5min in TBS before secondary antibodies incubation. For 1.5h at RT, brain sections were incubated with donkey anti-mouse Alexa Fluor 555-conjugated (1:300 in TBS; cat# A31570, Invitrogen) and donkey anti-rabbit Alexa Fluor 647-conjugated (1:300 in TBS; cat# A31573, Invitrogen) antibodies, washed 5× 5min in PBS, mounted on slides and dried overnight. Slides were coverslipped in Fluoromount mounting medium.

For density and distribution analysis, six to eight rostra of the corpus callosum were imaged in a single plane at 20X using an Axio Imager M2 epifluorescence microscope equipped with an AxioCam MRm. For microglial morphology analysis, z-stacks of Iba1^+^/TMEM119^+^ cells (17–22 ​cells/animals, n ​= ​5 animals/diet/sex) were captured at 40X using a Quorum Wave FX spinning disc confocal microscope (Quorum Technologies, Canada) equipped with an ORCA-R2 camera (512 ​× ​512 pixels; Hamamatsu Photonics, Hamamatsu, Japan) and merged into a single plane using Volocity software (Version 5.4, PerkinElmer, Waltham, MA, United States). All analyses were performed in double-blind using ImageJ. Counts of Iba1^+^/TMEM119^+^ (resident microglia) and Iba1^+^/TMEM119^-^ (peripheral myeloid cells) cells (Bennett et al., 2016) were performed as described (Tremblay et al., 2012; Ibanez et al., 2019) using the “Analyze Particles” plugin, and the rostrum area was traced using the “Freehand” tool to determine cell density. Microglial morphological index was calculated to reveal microglial changes from their steady-state (Tremblay et al., 2012; Ibanez et al., 2019) using the following formula:

$$Morphological\;Index=\;\frac{Soma\;area\;}{Arborization\;area}$$

For morphology analysis, soma and manual arbor were traced using the “Freehand” tool and “Polygon Selection” tool to obtain area and perimeter values. Manual arbor selection was processed in a semi-automatic manner (Bordeleau et al., 2020), using a method adapted from (Young and Morrison, 2018), to obtain masks of the cells, adjusted by the observer when needed, and used to calculate cell area and shape descriptors of the cell (circularity, solidity, aspect ratio). The cell masks were further processed using the “Skeleton” plugin ​and analyzed with the “Skeleton 2D/3D” plugin. Skeleton analysis calculated number of branches, average length of branches, maximal branch length, as well as number of junctions.

### 2.3 Ultrastructural analysis

For ultrastructural analysis, animal cohort (n ​= ​4 animals/diet/sex) was transcardially perfused with ~15 ​mL of PBS, ~75 ​mL of 3.5% acrolein in [100 ​mM] PB (pH ​= ​7.4) and ~150 ​mL of 4% PFA. Brains were further post-fixed for 2h in 4% PFA, before being washed in PBS, and cut into coronal 50 ​μm-sections with a VT1200S vibratome (Leica Biosystem) and stored in cryoprotectant at −20 ​°C until use.

Two PFA/acrolein-perfused brain sections containing the rostrum of the corpus callosum (Bregma 1.42 ​mm; stereotaxic atlas of Paxinos and Franklin 4^th^ edition (Paxinos and Franklin, 2013)) were selected per animal (n ​= ​4 animals/diet/sex). Sections were washed 5× 5min in PBS then quenched in 0.3% H_2_O_2_ in PBS for 10min and permeabilized in 0.1% NaBH_4_ diluted in PBS for 30min. Sections were blocked 1h at RT in BB (10% fetal bovine serum, 3% bovine serum albumin, 0.01% Triton X-100 in [50 ​mM] TBS, pH ​= ​7.6) before incubation with primary antibody rabbit anti-Iba1 (1:1000 in BB; cat# 019–19741, FUJIFILM Wako Chemical, Osaka, Japan) overnight at 4 ​°C. The next day, brains sections were washed 5× 5min in TBS and incubated with secondary antibody biotinylated goat anti-rabbit (1:300 in TBS; cat# 111-066-046, Jackson ImmunoResearch, West Grove, PA, United States) for 1.5h at RT, followed by avidin-biotin complex (1:1:100 in TBS; cat# PK-6100,Vector Laboratories, Burlingame, CA, United States) for 1h at RT. Immunostaining was revealed in 0.05% diaminobenzidine (DAB, in TBS; cat# D5905-50TAB, MilliporeSigma) containing 0.015% H_2_O_2_ for 4.5min at RT. Excess DAB was washed out by rinsing 5× 5min in PB. Sections were then processed for electron microscopy (EM). Tissues were incubated in 3% ferrocyanide (in H_2_O; cat# PFC232.250, BioShop, Burlington, ON, Canada) combined (1:1) with 4% aqueous osmium tetroxide (cat# 19170, Electron Microscopy Sciences, Hatfield, PA, United States) for 1h, washed 5× 5min in PBS, incubated in 1% thiocarbohydrazide (in PBS; cat# 2231-57-4, Electron Microscopy Sciences) for 20min, washed 5× 5min in PBS, incubated in 2% osmium tetroxide (in H_2_O), then dehydrated in ascending concentration of ethanol (2× in 35%, 1× in 50%, 1× in 70%, 1× in 80%, 1× in 90%, 2× in 100%) followed by 3× in propylene oxide, for 5min each. After post-fixation, tissue sections were embedded in Durcupan ACM resin (cat# 44611-44614, MilliporeSigma) for 24h, placed between two ACLAR® embedding sheets (cat# 50425-25, Electron Microscopy Sciences) and resin was polymerised at 55 ​°C for 72h. Region of interest –the rostrum– was excised from the embedded sections on ACLAR® sheets and glued on top of resin blocks for ultrathin sectioning (Leica Ultracut UC7 ultramicrotome, Leica Biosystems).

Ultrathin sections (~70-75 ​nm of thickness) were collected and placed on copper 200-mesh grids for transmission EM imaging (myelin analysis), or on a silicon nitride chip and glued on specimen mounts for scanning EM imaging (microglial analysis). Samples collected on copper 200-mesh grids and used for myelin analysis were imaged using a FEI Tecnai Spirit G2 transmission EM operating at 80 ​kV and equipped with an ORCA-HR camera (10 ​MP; Hamamatsu Photonics). Samples collected on a silicon nitride chip and used for microglial analysis were imaged using a Zeiss Crossbeam 540 Gemini scanning EM (Zeiss), operating with an acceleration voltage of 1.4 ​kV and current of 1.2 ​nA.

#### 2.3.1 Myelin analysis

For each animal, 10 to 15 low-magnification images of the rostrum were acquired at 2900X to quantify myelinated axon density (n ​= ​4 animal/diet/sex) and one representative low-magnification area was selected randomly for the creation of a mosaic composed of 14–19 higher-magnification images at 9300X to evaluate myelinated axon g-ratio and quantify cytoplasmic channels (n ​= ​302–436 axons/diet/group, N ​= ​4 animals/group). All analyses were performed blind to the experimental conditions. Using ImageJ, the “Cell Counter” plugin was used to count the number of myelinated axons. For density analysis, all incomplete axons where not considered. Cytoplasmic channels recognized as clear cytoplasmic content within the myelin sheaths (Snaidero et al., 2017) with an area over 1000 ​nm^2^ were counted. Their area was traced using the “Freehand” tool and measured. To calculate the g-ratio for each axon, axon (without myelin sheath) and myelinated axon (including the axon and its myelin sheath) areas were delineated using the “Freehand” tool separately, then the axon and myelinated axon diameters were determined using “Feret's Diameter” plugin. G-ratio was calculated using the following formula:

$$\text{g-ratio}=\frac{Diameter_{inner}}{Diameter_{outer}}$$

Tracing of myelinated axon was also used to determine shape descriptors (circularity, solidity, roundness, aspect ratio).

#### 2.3.2 Microglial analysis

Microphotographs of seven to 11 microglial cell bodies (n ​= ​33–41 microglia/diet/sex, N ​= ​4 animal/group) were acquired at 5 ​nm resolution (x,y). All analyses were performed blind to the experimental conditions. For microglial ultrastructure analyses, organelles (i.e., endosomes, endoplasmic reticulum, Golgi apparatus, lipid bodies, lysosomes, lipofuscins and mitochondria) and their anomalies (i.e., dilation of endoplasmic reticulum and Golgi apparatus cisternae) (Chavez-Valdez et al., 2016; Hajj et al., 2019) within microglial cell bodies were quantified on a cellular basis. Number of microglial cell bodies contacting other cells (i.e., astrocytes, dark cells, microglia, oligodendrocytes), neuronal elements (i.e., myelinated axons and synapses –presynaptic axon terminals and postsynaptic dendritic spines), extracellular digestion activities (degenerating myelin, extracellular digestion), blood vessels, and extracellular space pockets were also quantified on a cellular basis as previously described (Bisht et al., 2016; Chavez-Valdez et al., 2016; Hajj et al., 2019; St-Pierre et al., 2019; Lecours et al., 2020; Savage et al., 2020).

Lysosomes were recognized by their electron-dense heterogenous circular structure and divided into primary (De Duve, 1963; Holtzman et al., 1967), secondary (associated with endosomes) and tertiary (associated with lipofuscins and often with endosomes) categories (De Duve, 1963; Nandy, 1971). Lipofuscins were recognized by their electron-dense spherical structure with a distinct fingerprint-like pattern (Nandy, 1971). Endoplasmic reticulum and Golgi apparatus cisternae were considered dilated when the space between their two membranes was 50 ​nm or greater. Elongated mitochondria were identified by a length of 1 ​μm or greater (St-Pierre et al., 2019). Astrocytic cells were identified by their pale nuclei with a thin rim of heterochromatin and their pale irregular cytoplasm, often containing intermediate filaments (Peters et al., 1991). Neurons were distinguished by their pale nuclei and pale cytoplasm, often with an apical dendrite and synaptic contacts (Peters et al., 1991). Microglia were recognized by their immunoreactivity to Iba1 as well as ultrastructural features including: their dark irregular nuclei with a heterogenous chromatin pattern and dark irregular cytoplasm, often containing long stretch of endoplasmic reticulum cisternae and lipidic inclusions (i.e., lipofuscins, lipid bodies, lipid droplets and lysosomes) (Peters et al., 1991). Oligodendrocytes were distinguished by their dark round or oval nuclei with a heterogenous chromatin pattern and their dark squared-shape wide cytoplasm containing short, wide endoplasmic reticulum cisternae and often enriched in ribosomes (Peters et al., 1991). Oligodendrocyte progenitor or precursor cells were differentiated from other glial cells by their pale irregular nuclei and angular-shaped cytoplasm containing small mitochondria but no intermediate filaments (Peters et al., 1991). Synapses were identified by a visible synaptic density, where presynaptic elements were differentiated from postsynaptic elements by the presence of synaptic vesicles (Peters et al., 1991). Extracellular digestion or digestive “exophagy” was identified by extracellular space pockets containing degraded elements or debris in areas directly adjacent to the microglial cell body (Haka et al., 2016; Acharjee et al., 2018; Maxfield et al., 2020). Degenerating myelin was recognized by ballooning, swelling or distancing between the well-defined myelin sheaths (Peters et al., 1991). Microglial contacts with capillaries were counted when microglial cell bodies directly touched their basement membrane, which forms a thin electron-dense layer encompassing the capillary's cells including endothelial cells, pericytes and other perivascular cells.

### 2.4 Transcriptomic analysis

For transcriptomic analysis, the animals (n ​= ​5–6 animals/diet/sex) were anesthetized with 100 ​mg/mL ketamine/20 ​mg/mL xylazine/10 ​mg/mL acepromazine rodent cocktail (0.3 ​mL/100g), freshly-decapitated, their brain extracted, and regions of interest were dissected then flash-frozen on dry ice and kept at −80 ​°C until RNA extraction. Due to technical limitation in microdissecting confidently the adolescent corpus callosum, we focussed on projecting regions of the anterior corpus callosum when selecting the regions of interest.

Regions of interest (hippocampus and prefrontal cortex) from each animal were homogenized in Trizol (cat# 15596-026, Ambion, Austin, TX, United States) and their RNA was extracted using the Trizol/chloroform method followed by an isopropanol precipitation. RNA pellet was washed once in 75% ethanol, let dry, then eluted in nuclease-free water (cat# AM9937, Ambion). Samples were dosed using the NanoDrop ND-1000 (Thermo Fisher Scientific).

Genomic DNA was removed from 1 ​μg isolated RNA sample by enzymatic degradation (cat# G488, Applied Biological Materials Inc, Richmond, BC, Canada). Purified RNA was used to obtain complementary DNA (cDNA) by reverse transcriptase reaction with iScript 5X MasterMix (cat# 1708890, BioRad Laboratories, Hercules, CA, United States) using a TI thermocycler (Biometra, Göttingen, Germany). Using diluted cDNA, rt-qPCR was performed with the SybrGreen technology and cycle threshold (Ct) was determined using a LightCycler 480 II (Roche, Basel, Switzerland). Rt-qPCR measuring transcripts of microglial function-related genes *Mbp*, *Plp1, Olig2, Cspg4* (coding for NG2)*, Gmpb, Igf1* as well as housekeeping gene *Rpl32* was run (Table 1). Relative expression was calculated by the following formulas while considering CD-exposed male offspring as the reference group:

$$\text{ΔCt}={\text{Ct}}_{experimental\;group}-{\text{ ​Ct}}_{reference\;group\;\left(CD\;male\right)}$$

$$\text{ΔΔCt}={\text{Ct}}_{target}-{\text{Ct}}_{Rpl32}$$

$$\text{Relative ​expression}=2^{-\text{ΔΔCt}}$$

**Table 1:** Primer pairs of myelination-related genes (*Mbp*, *Plp1*, *Cspg4, Olig2*), myelination factors expressed by microglia (*Igf1* and *Gpnmb*) and housekeeping gene *Rpl32* evaluated by RT-qPCR. *Cspg4*: chondroitin sulfate proteoglycan 4, *Gpnmb*: glycoprotein Nmb, *Igf1*: insulin-like growth factor 1, *Mbp*: myelin basic protein, *Olig2*: oligodendrocyte factor 2, *Plp1*: proteolipid protein 1, *Rpl32*: ribosomal protein L32.

Gene	5′primer	3′primer
**Myelination-related genes**		
*Mbp*	CCC TCA CAG CGA TCC AAG TA	TAA AGA AGC GCC CGA TGG A
*Plp1*	GGC TCC AAC CTT CTG TCC AT	TCG GCC CAT GAG TTT AAG GA
*Cspg4* (NG2)	AGA TCC TCC ACA ACA CAG GG	GGT ACC AGT GAC TCG GAA CA
*Olig2*	GCG CGA AAC TAC ATC CTC AT	CGT AGA TCT CGC TCA CCA GT
**Myelination factor genes expressed by microglia**		
*Igf1*	GGC ATT GTG GAT GAG TGT TG	GTC TTG GGC ATG TCA GTG TG)
*Gpnmb*	AAA ACT GGG TCG GTG TTC AG	CAC CTT CGA GAT GGG AAT GT
**Housekeeping gene**		
*Rpl32*	TTG TTG CTC CCA TAA CCG ATG	TTA AGC GAA ACT GGC GGA AAC

Results were presented in ratio fold and statistical analysis was assessed on ΔΔCt that are normally distributed, as previously conducted (Hui et al., 2018; Bordeleau et al., 2020).

### 2.5 Offspring behavioral assessment

In adolescence (P32–P40), we assessed social memory (n ​= ​6–8 litters/diet/sex, N ​= ​6–11 animals/group), anxiety-related behaviors (n ​= ​6–8 litters/diet/sex, N ​= ​6–11 animals/group) and sensorimotor gating (n ​= ​7–10 litters/diet/sex, N ​= ​8–12 animals/group) of the offspring exposed to CD or mHFD. Prior to each behavioral test, the animals were acclimated to the experimental room and conditions for 30min. All behaviors were assessed in a way that prevented the animals from seeing the experimenter throughout the tests. Between each trial and test, the arena and objects were cleaned using a mixture of ethanol/peroxide to prevent olfactory cues.

#### 2.5.1 Social interaction

For two days prior to testing, intruder mice were acclimated to the wired cage under infrared light for 20min to minimize the stress of the animals during social interaction assessment.

Social preference and social memory (also referred to as social novelty preference) were measured with the three-chambers social interaction paradigm under infrared light during the active phase of the animals (20:00–23:59). For 10min, experimental animals were habituated to the 3-chambers arena with the two wired cages placed inside the side compartments (chamber: 26 ​cm (l) ​× ​21.6 ​cm (w) ​× ​21.6 ​cm (h); door: 5 ​cm (w) ​× ​5 ​cm (h)). After the habituation, an intruder mouse was randomly placed in one of the two wired cages (dimension: 7.6 ​cm (d) ​× ​9.5 ​cm (h)), and a toy was placed in the other wired cage. Social preference was assessed for 10min, then the toy was substituted with an unknown intruder to evaluate social novelty preference for another 10min session.

All test sessions were filmed, then scored automatically using the tracking software TopScan Version 2.00 (Clever Sys Inc, Reston, VA, United States). Time spent and number of entries in each compartment, as well as contacts/sniffing time with the occupied wired cage were measured using TopScan software. Social preference and social memory indices were respectively calculated by the ratio of time interacting with intruder over toy, and time interacting with novel over familiar intruders.

#### 2.5.2 Elevated plus maze

Anxiety was gauged with the elevated plus maze test. Elevated plus maze was assessed at the beginning of the light phase (8:00–11:00) under normal housing lighting (~30 lux). Mice were placed at the junction of the open and closed arms facing the open arms and let free to explore the elevated plus maze (open arm: 29.25 ​cm (l) ​× ​5 ​cm (w); closed arm: 29.25 ​cm (l) ​× ​5 ​cm (w) ​× ​11.5 ​cm (h); center: 5 ​cm (w) ​× ​5 ​cm (l)) for 10min. After the 5min session, the animal was returned to its home cage.

For each animal, test sessions were filmed for later analysis using TopScan software. Time spent and number of entries in open arms, closed arms and at the center of the maze were determined to evaluate anxiety-related behaviors. An entry was considered when three paws were detected in one of the designated zones (open arm, closed arm, or center).

#### 2.5.3 Prepulse inhibition

Prepulse inhibition (PPI) is the reduction of the startle response to a strong auditory stimulus when the stimulus is preceded by a weaker stimulus that is known as sensorimotor gating (Mena et al., 2016). Alterations of PPI are often observed in neurodevelopmental disorders like schizophrenia (Mena et al., 2016).

Sensorimotor gating was evaluated using a commercially available system allowing to perform PPI of the acoustic startle reflex (SR-LAB; San Diego Instruments, San Diego, CA, United States). Animals were placed inside a cylindrical Plexiglas animal enclosure within the experimental box. After 5min of habituation, 42 discrete trials were performed; the first two trials were 120 ​dB in magnitude (startle pulse) followed by 40 trials with a randomly assigned order, constituting of 10 trials of the startle alone, 5 trials where a specific 30 ​ms prepulse (3 ​dB, 6 ​dB, 9 ​dB, 12 ​dB or 15 ​dB) occurred 100 ​ms prior the startle, and 5 trials without a prepulse and startle cue (null response). Startle responses were automatically determined by the SR-LAB system. PPI was calculated as the relative percentage of the mean amplitude of the startle response without prepulse (startle pulse alone) compared to responses recorded after a 30 ​ms prepulse, using the following formula:

$$\text{PPI}=100-\left(\frac{{\text{Mean}}_{prepulse}}{{\text{Mean}}_{startle}}\right)\times 100$$

### 2.6 Statistical analyses

All statistical analyses were conducted using Prism 8 (v.8.4.2, GraphPad Software, San Diego, CA, United States) and results presented as mean ​± ​standard error of the mean (SEM). Normality was verified with a Shapiro-Wilk test. For normally distributed dataset, Grubbs’ test (two-tailed, α ​= ​0.05) was used to remove outliers from the datasets prior to conducting parametric analyses. Two-way ANOVAs were used for all analyses, except for microglial ultrastructure analyses, to compare variance difference between *Diet* (CD *vs* mHFD), *Sex* (male *vs* female) as well as *Diet∗Sex* interaction. Significant ANOVA tests with a *Diet∗Sex* interaction were followed by a Bonferroni *post-hoc* test to identify significant differences between all groups. Mixed-effect model followed by a Bonferroni *post-hoc* test was used for the non-normally distributed dataset of microglial ultrastructure analyses. Significance was reached when *p* value was lower than 0.05.

## 3 Results

### 3.1 Changes in myelin of the corpus callosum and myelination-related hippocampal transcripts of mHFD-exposed adolescent male offspring

**Fig. 1:**
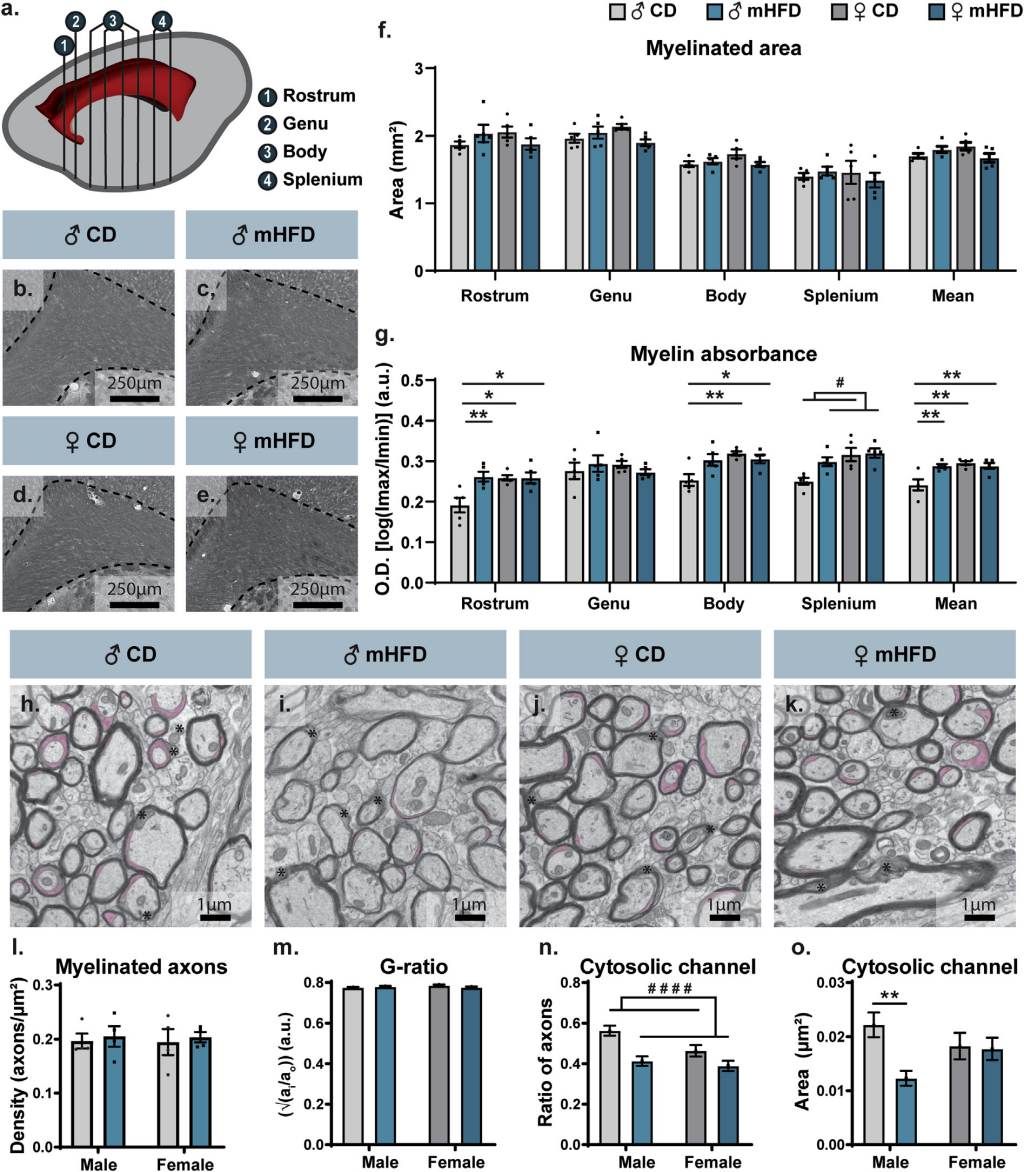
Increased integrated density of corpus callosum of P30 male offspring associated to reduced area of myelin cytosolic channels. (a) Scheme illustrates sections of the corpus callosum used to assess (1: rostrum; 2: genu; 3: body; 4: splenium). (b–e) Representative pictures of the rostrum of the corpus callosum stained by Sudan Black B, where the corpus callosum is delimitated by black dashed lines. (f) Area and (g) optical density were measured for each section of the corpus callosum (8–9 sections/animal, n ​= ​5 animals/group). (h–k) Representative pictures of the rostrum ultrastructure showing myelinated axon density, g-ratio proportion, and cytoplasmic channel prevalence (pseudocolored in pink) in the different groups. (l) Myelinated axon density was determined for every animal (10–15 images/animal, n ​= ​4 animals/group). (m) G-ratio and (n–o) cytoplasmic channel prevalence were then assessed for each high-magnified axon of each individual animal (n ​= ​302–436 axons/group, N ​= ​4 animals/group). Graphs represent Mean ​± ​SEM. Statistical significance was determined by 2-way ANOVA followed by Bonferroni *post-hoc* analysis, where ∗ *p* ​< ​0.05, ∗∗*p* ​< ​0.01 for *Diet∗Sex effect*, ^#^*p* ​< ​0.05, ^# # # #^*p* ​< ​0.0001 for *Diet effect*. ♂: male, ♀: female, CD: control diet, mHFD: maternal high-fat diet. (For interpretation of the references to color in this figure legend, the reader is referred to the Web version of this article.)

Considering recent findings that associate mHFD with altered myelination in the medial cortex of P21 mouse offspring (Graf et al., 2016), we investigated myelination changes within the corpus callosum using Sudan Black B at the timepoint at which fibers of the corpus callosum reach maturity, P30 (Baloch et al., 2009). The corpus callosum represents the main collection of fiber projections within the brain, making it critical for neuronal influx propagation, as well as communication between brain regions (Fig. 1a) (Goldstein et al., 2020). Sudan Black B staining analysis showed no significant change in myelin area coverage of the different regions of the corpus callosum (rostrum, genu, body and splenium) (Fig. 1 b-f). However, assessment of myelin optical density –an indicator of changes in myelin organization or integrity– showed globally increased optical density levels in the mHFD-exposed male compared to female offspring (male CD *vs* male mHFD: *p* ​= ​0.0081, *vs* female CD: *p* ​= ​0.0023*,* *vs* female mHFD: *p* ​= ​0.0082; Fig. 1b–e,g, Supplementary Table 1). Specifically, this significant difference was most striking in the rostrum, the corpus callosum part that comprises mainly the interhemispheric projections of the anterior areas of the cerebral cortex [i.e., prefrontal, premotor, motor and associative areas (Raybaud, 2010; Goldstein et al., 2020)] (male CD *vs* male mHFD: *p* ​= ​0.0097, *vs* female CD: *p* ​= ​0.0137*,* *vs* female mHFD: *p* ​= ​0.0143; Fig. 1b–e,g, Supplementary Table 1). No significant difference was measured in the genu. Nevertheless, myelin optical density was higher in the body of CD-exposed female compared to male offspring (male CD *vs* female CD: *p* ​= ​0.0068*,* *vs* female mHFD: *p* ​= ​0.0385), while optical density was increased in the splenium of mHFD-exposed offspring compared to control offspring regardless of their sex, and of females compared to males regardless of their maternal diet exposure (Fig. 1b–e,g, Supplementary Table 1).

We next proceeded with a thorough ultrastructural characterization of myelinated axons in P30 offspring of the two sexes using EM, focusing on the region of the corpus callosum that was the most altered at the light level, the rostrum, to understand how the organization of myelin sheaths might be impacted. No significant change in myelinated axon density, g-ratio, and decompacted myelin was observed (Fig. 1h-m, Table 2). However, the ratio of myelinated axons possessing cytosolic myelin channels, channels formed by mature oligodendrocytes that are hypothesized to provide for the metabolic needs of neurons and support the axon-myelin unit (Snaidero et al., 2017), was decreased in male and female offspring after exposure to mHFD (Fig. 1h-m, Table 2). Furthermore, only mHFD-exposed male offspring showed a reduced area of these channels compared to their CD-exposed male counterpart (male CD *vs* male mHFD: *p* ​= ​0.0016; Fig. 1h-m, Table 2). Shape descriptors of myelinated axons additionally revealed significant changes in axonal morphology upon mHFD (Table 2). Indeed, circularity and roundness values were reduced in mHFD-exposed compared to control offspring regardless of their sex, while their values were greater in male compared to female offspring regardless of their diet (Table 2). All offspring groups also presented significantly different values of aspect ratio (male CD *vs* male mHFD *vs* female CD *vs* female mHFD: *p* ​< ​0.0001, for all *post-hoc* tests; Table 2). Of note, axons of the corpus callosum did not show ultrastructural signs of cellular stress or degeneration, i.e., darker cytoplasm (see Fig. 1h–k). Overall, the decrease in cytosolic myelin channels provides an explanation for the increased optical density observed at the light level, while the morphometric changes of myelinated axons could indicate differences in axonal function and conductivity adaptability (Arbuthnott et al., 1980).

**Table 2:** Characterization of myelinated axons in the rostrum of the corpus callosum of P30 offspring exposed to mHFD. 10–15 images/animal, n ​= ​4 animals/group (density); n ​= ​302–436 axons/group, N ​= ​4 animals/group (g-ratio, cytosolic channels, decompacted myelin and shape descriptors). Statistical significance was determined by 2-way ANOVA followed by Bonferroni *post-hoc* analysis, where ∗ *p* ​< ​0.05, ∗∗∗*p* ​< ​0.001, ∗∗∗∗*p* ​< ​0.0001. ♂: male, ♀: female, a.u.: arbitrary unit, CD: control diet, mHFD: maternal high-fat diet.

Parameters		Mean ​± ​standard error of the mean				*F*	*p*
		Male		Female			
		CD	mHFD	CD	mHFD		
Density (axons/μm^2^)		0.196 ​± ​0.014	0.205 ​± ​0.019	0.195 ​± ​0.024	0.203 ​± ​0.010	Sex∗Diet: 0.0001575 Sex: 0.009132 Diet: 0.2451	Sex∗Diet: 0.9902 Sex: 0.9254 Diet: 0.6295
G-ratio (a.u.)		0.775 ​± ​0.004	0.780 ​± ​0.004	0.786 ​± ​0.005	0.776 ​± ​0.005	Sex∗Diet: 2.878 Sex: 0.8895 Diet: 0.3245	Sex∗Diet: 0.0900 Sex: 0.3458 Diet: 0.5690
Cytosolic channels	Ratio (a.u.)	0.563 ​± ​0.025	0.413 ​± ​0.024	0.464 ​± ​0.029	0.389 ​± ​0.025	Sex∗Diet: 2.165 Sex: 5.825 Diet: 19.33	Sex∗Diet: 0.1414 Sex: **0.0159∗** Diet: **< 0.0001∗∗∗∗**
	Area (μm^2^)	0.0222 ​± ​0.002	0.0123 ​± ​0.001	0.0183 ​± ​0.002	0.0177 ​± ​0.002	Sex∗Diet: 5.293 Sex: 0.1385 Diet: 6.532	Sex∗Diet: **0.0215∗** Sex: 0.7098 Diet: **0.0107∗**
Ratio decompacted myelin (a.u.)		0.341 ​± ​0.024	0.360 ​± ​0.023	0.358 ​± ​0.028	0.357 ​± ​0.025	Sex∗Diet: 0.1689 Sex: 0.07284 Diet: 0.1367	Sex∗Diet: 0.6812 Sex: 0.7873 Diet: 0.711
Shape descriptors of myelinated axons	Circularity (a.u.)	0.809 ​± ​0.005	0.783 ​± ​0.004	0.771 ​± ​0.006	0.762 ​± ​0.005	Sex∗Diet: 2.365 Sex: 32.27 Diet: 11.68	Sex∗Diet: 0.1243 Sex: **< 0.0001∗∗∗∗** Diet: **0.0006∗∗∗**
	Solidity (a.u.)	0.977 ​± ​0.008	0.974 ​± ​0.001	0.975 ​± ​0.001	0.975 ​± ​0.001	Sex∗Diet: 3.077 Sex: 0.4825 Diet: 1.518	Sex∗Diet: 0.0796 Sex: 0.4874 Diet: 0.2181
	Roundness (a.u.)	0.682 ​± ​0.001	0.611 ​± ​0.007	0.608 ​± ​0.010	0.565 ​± ​0.007	Sex∗Diet: 3.311 Sex: 56.28 Diet: 50.52	Sex∗Diet: 0.1266 Sex: **< 0.0001∗∗∗∗** Diet: **< 0.0001∗∗∗∗**
	Aspect ratio (a.u.)	1.586 ​± ​0.027	1.737 ​± ​0.023	0.786 ​± ​0.036	1.895 ​± ​0.027	Sex∗Diet: 399.9 Sex: 179.7 Diet: 690.9	Sex∗Diet: **< 0.0001∗∗∗∗** Sex: **< 0.0001∗∗∗∗** Diet: **< 0.0001∗∗∗∗**

**Fig. 2:**
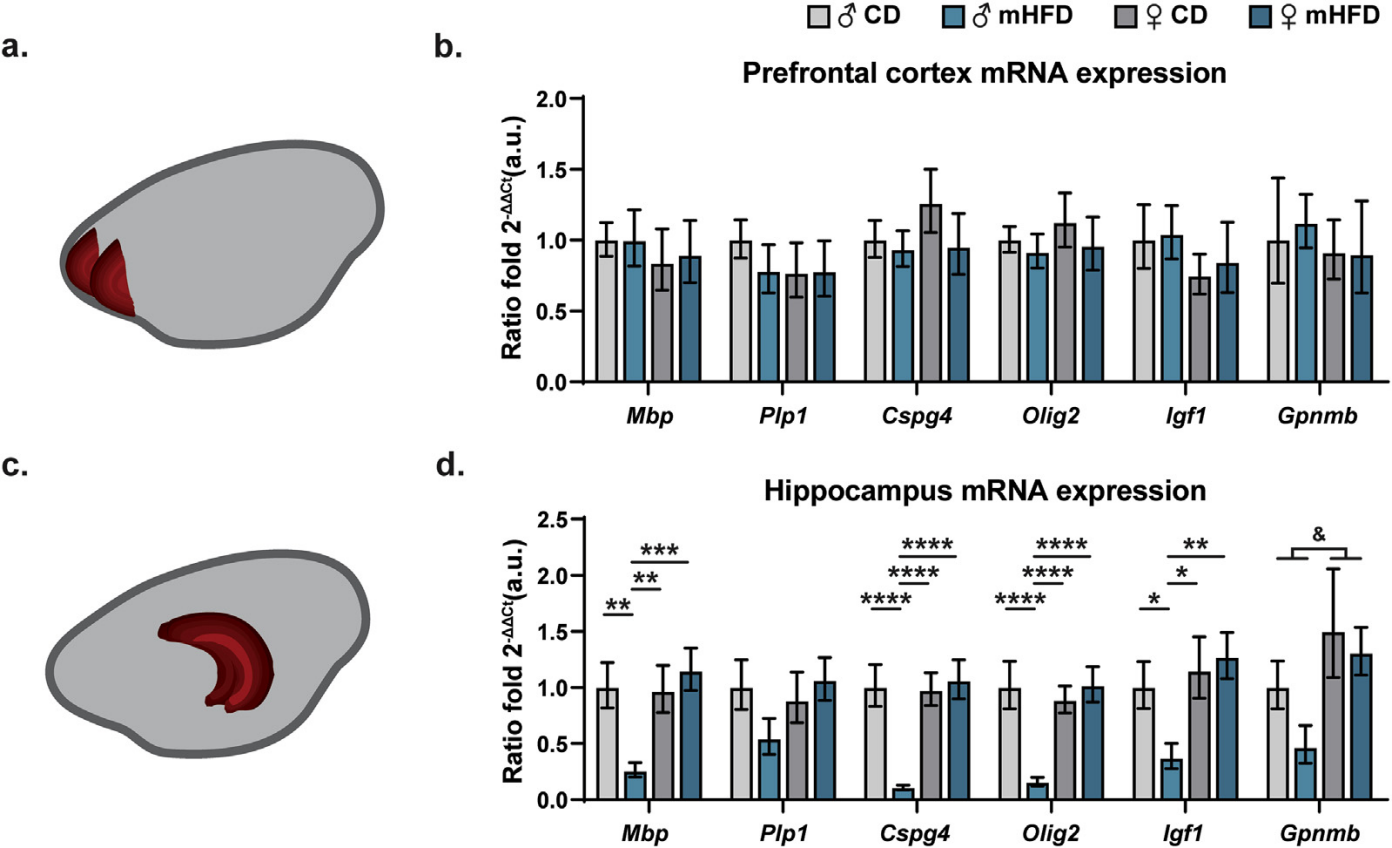
Differential alteration of myelin-related genes in projecting regions of the rostrum of the corpus callosum, the prefrontal cortex and hippocampus, in P30 male offspring. Schemes illustrate regions of interest: (a) prefrontal cortex and (c) hippocampus. *Mbp*, *Plp1*, *Cspg4* (NG2), *Olig2*, *Igf1b* and *Gpnmb* mRNA expression levels were quantified in the (b) prefrontal cortex and (d) hippocampus (n ​= ​5–6 animals/group). Bar graphs represent calculated Mean of ratio fold 2^ΔΔCt^ ​± ​calculated SEM. Statistical significance was assessed by 2-way ANOVA followed by Bonferroni *post-hoc* analysis, where ∗ *p* ​< ​0.05, ∗∗*p* ​< ​0.01, ∗∗∗*p* ​< ​0.001, ∗∗∗∗*p* ​< ​0.0001 for *Diet∗Sex effect*, ^&^*p* ​< ​0.05 for *Sex effect*. ♂: male, ♀: female, CD: control diet, *Cspg4*: chondroitin sulfate proteoglycan 4, *Gpnmb*: glycoprotein Nmb, *Igf1*: insulin-like growth factor 1, *Mbp*: myelin basic protein, mHFD: maternal high-fat diet, *Olig2*: oligodendrocyte factor 2, *Plp1*: proteolipid protein 1, *Rpl32*: ribosomal protein L32.

To further characterize the myelination changes, we measured the expression of genes associated with myelination in regions with projecting axons located in the anterior part (rostrum and genu) of the corpus callosum, i.e., the prefrontal cortex (Fig. 2a) and the hippocampus (Fig. 2c) (Raybaud, 2010; Goldstein et al., 2020). We found no significant difference in the prefrontal cortex, however, in the hippocampus, several transcripts, including *Mbp*, *Cspg4*, *Olig2*, and *Igf1*, were significantly lower in male offspring exposed to mHFD compared to other offspring groups (*Mbp*: male mHFD *vs* male CD: *p* ​= ​0.0014, *vs* ​female CD: *p* ​= ​0.0018*, vs* ​female mHFD: *p* ​= ​0.0005, *Cspg4*: male mHFD *vs* male CD: *p* ​< ​0.0001, *vs* ​female CD: *p* ​< ​0.0001*, vs* ​female mHFD: *p* ​< ​0.0001, *Olig2*: male mHFD *vs* male CD: *p* ​< ​0.0001, *vs* ​female CD: *p* ​< ​0.0001*, vs* ​female mHFD: *p* ​< ​0.0001, *Igf1*: male mHFD *vs* male CD: *p* ​= ​0.0412, *vs* female CD *p* ​= ​0.0159*, vs* female mHFD *p* ​= ​0.0081; Fig. 2b,d). Furthermore, *Gpnmb* expression was greater in female offspring compared to male offspring regardless of their maternal diet (Fig. 2d, Supplementary Table 2). Together, these results suggest that mHFD-exposed male offspring show altered myelination of their hippocampus during adolescence, highlighting that the myelin changes of the corpus callosum may impact on neuronal network connectivity.

### 3.2 Changes in myelination are not associated to oligodendrocytes density, distribution, and maturation differences in the corpus callosum of mHFD-exposed adolescent offspring

**Fig. 3:**
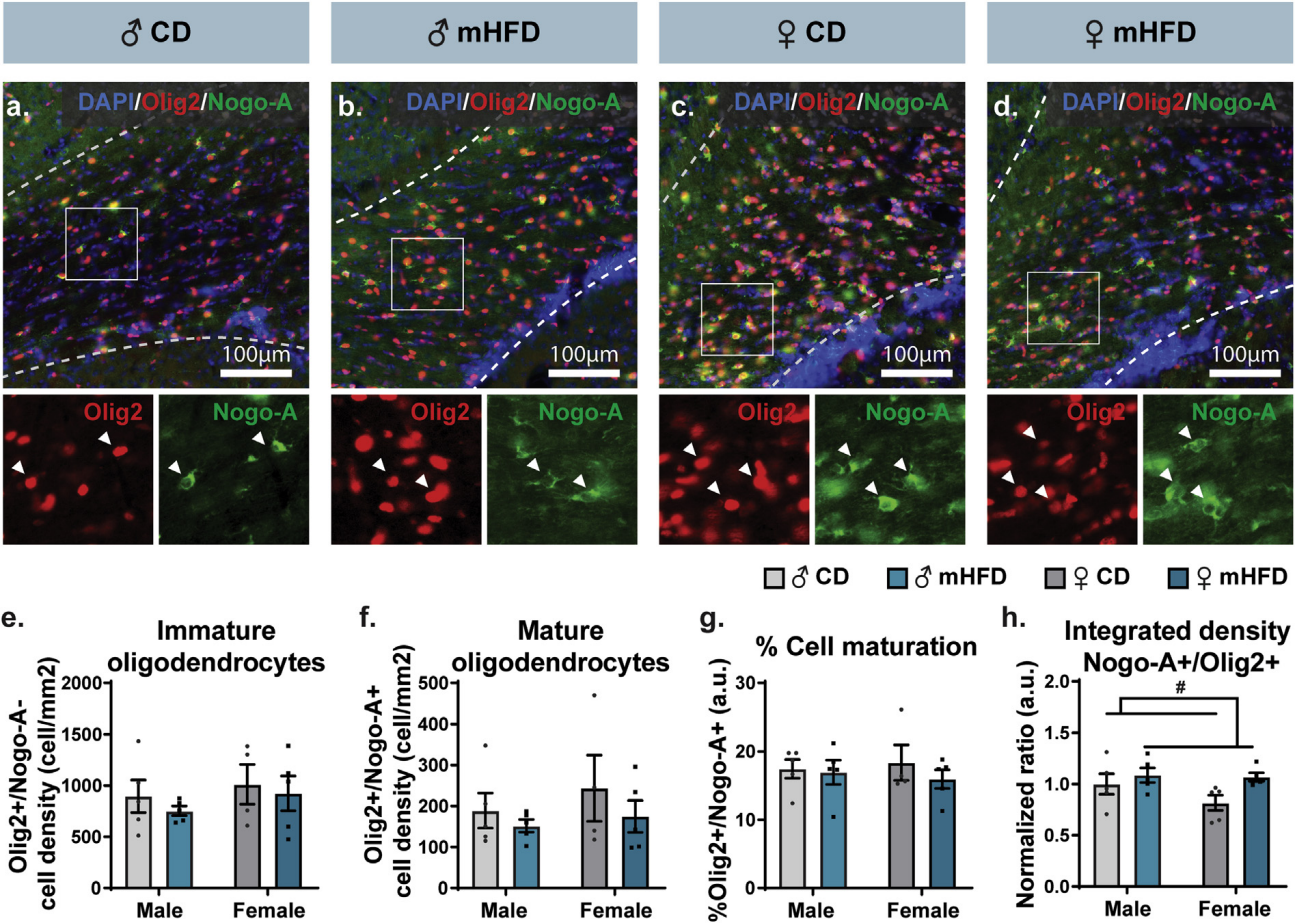
Cytosolic myelin channel changes are not attributed to oligodendrocytic maturation in P30 male offspring. (a–d) Representative pictures of Olig2(red)/Nogo-A(green)/DAPI(blue) immunofluorescence are provided for each offspring group, where close-up insets (100 ​μm ​× ​100 ​μm square) show colocalization of Olig2 (left side in red) and Nogo-A (right side in green) identifying mature oligodendrocytes. In representative pictures, delimitation of the rostrum is shown by white dashed lines. Density, distribution, and maturation of oligodendrocytes were determined. Main results are represented, including (e) immature (Olig2^+^/Nogo-A^-^) and (f) mature (Olig2^+^/Nogo-A^+^) oligodendrocytes density, (g) percentage of cell maturation, and (h) ratio of Nogo-A/Olig2 fluorescence intensity (3–4 sections/animal, n ​= ​5 animals/group). Statistical significance was determined by 2-way ANOVA, where ^#^*p* ​< ​0.05 for *Diet effect*. Bar graphs represent Mean ​± ​SEM. ♂: male, ♀: female, CD: control diet, mHFD: maternal high-fat diet. (For interpretation of the references to color in this figure legend, the reader is referred to the Web version of this article.)

Ultrastructural analysis identified a reduction of cytosolic myelin channels prevalence and area in the rostrum of the corpus callosum, specifically in P30 male offspring exposed to mHFD. Impairment in these metabolic channels of the axon-myelin unit has been previously reported to result from a reduced oligodendrocyte maturation. In particular, these cells are the main regulators of the balance between myelin basic protein (MBP) and 2′,3′-*cyclic*-nucleotide 3′-phosphodiesterase (CNP), expressed as an MBP/CNP ratio, which influences the formation of cytosolic myelin channels (Snaidero et al., 2017). We therefore sought to appraise oligodendrocyte maturation within the rostrum of P30 offspring of the two sexes, using immunofluorescence staining to visualize total, immature and mature oligodendrocyte populations (Fig. 3). Analysis of total (Olig2^+^), immature (Olig2^+^/Nogo-A^-^) and mature oligodendrocyte (Olig2^+^/Nogo-A^+^) population density, as well as rate of cell maturation (mature oligodendrocyte/total oligodendrocytes) showed no significant difference between offspring groups (Fig. 3a–g, Table 3). Fluorescence intensity, measured by the integrated density of Olig2 immunostaining, was reduced in mHFD-exposed compared to control offspring regardless of their sex, while Nogo-A immunostaining remained unchanged between groups (Table 3), leading to a slight increase of Nogo-A/Olig2 integrated density ratio in the mHFD-exposed offspring (Fig. 3h, Table 3). Together, these results suggest an absence of maturation impairment within oligodendrocyte populations, where both immature and mature populations showed similar number/density to control groups in males and females.

**Table 3:** mHFD effects on oligodendrocytes population density, distribution and maturation, as well as fluorescence intensity of Olig2 and Nogo-A within the rostrum of the corpus callosum of P30 offspring. 3–4 sections/animal, n ​= ​5 animals/group. Statistical significance was determined by 2-way ANOVA, where ∗ *p* ​< ​0.05. ♂: male, ♀: female, a.u.: arbitrary unit, CD: control diet, mHFD: maternal high-fat diet.

Parameters		Mean ​± ​standard error of the mean				*F*	*p*
		Male		Female			
		CD	mHFD	CD	mHFD		
Density (cells/mm^2^)	Olig2^+^	1085 ​± ​199	905.0 ​± ​44.8	1256 ​± ​260.7	1099 ​± ​203.9	Sex∗Diet: 0.003761 Sex: 0.9492 Diet: 0.8141	Sex∗Diet: 0.9519 Sex: 0.3454 Diet: 0.3812
	Olig2^+^/Nogo-A^-^	1085 ​± ​199	905.0 ​± ​44.8	1256 ​± ​260.7	1099 ​± ​203.9	Sex∗Diet: 0.003761 Sex: 0.9492 Diet: 0.8141	Sex∗Diet: 0.9519 Sex: 0.3454 Diet: 0.3812
	Olig2^+^/Nogo-A^+^	189.1 ​± ​42.5	152.0 ​± ​15.4	243.7 ​± ​80.5	174.8 ​± ​38.9	Sex∗Diet: 0.1202 Sex: 0.7094 Diet: 1.331	Sex∗Diet: 0.7336 Sex: 0.4129 Diet: 0.2666
Spacing index (a.u.)	Olig2^+^	0.356 ​± ​0.018	0.339 ​± ​0.003	0.341 ​± ​0.017	0.343 ​± ​0.009	Sex∗Diet: 0.4879 Sex: 0.1592 Diet: 0.3077	Sex∗Diet: 0.4949 Sex: 0.6952 Diet: 0.5868
	Olig2^+^/Nogo-A^+^	0.175 ​± ​0.030	0.158 ​± ​0.42	0.156 ​± ​0.024	0.151 ​± ​0.022	Sex∗Diet: 0.04033 Sex: 0.1857 Diet: 0.1264	Sex∗Diet: 0.8434 Sex: 0.6723 Diet: 0.7268
Integrated density (a.u.)	Olig2^+^	46454 ​± ​4443	37809 ​± ​3116	45577 ​± ​3186	36844 ​± ​2990	Sex∗Diet: 0.0001597 Sex: 0.06978 Diet: 6.220	Sex∗Diet: 0.9901 Sex: 0.7950 Diet: **0.0240∗**
	Nogo-A^+^	57635 ​± ​5100	61180 ​± ​3859	69984 ​± ​2259	62973 ​± ​1497	Sex∗Diet: 2.310 Sex: 4.145 Diet: 0.2489	Sex∗Diet: 0.1481 Sex: 0.0587 Diet: 0.6246
Maturation	% cell density	17.45 ​± ​1.37	16.96 ​± ​1.78	18.36 ​± ​2.60	15.93 ​± ​1.36	Sex∗Diet: 0.3038 Sex: 0.001173 Diet: 0.6846	Sex∗Diet: 0.5896 Sex: 0.9731 Diet: 0.4210
	Nogo-A^+^/Olig2^+^ integrated density (a.u.)	1.000 ​± ​0.101	1.086 ​± ​0.071	0.816 ​± ​0.074	1.070 ​± ​0.040	Sex∗Diet: 1.251 Sex: 1.803 Diet: 5.175	Sex∗Diet: 0.2798 Sex: 0.1981 Diet: **0.0370∗**

### 3.3 Altered microglial phagolysosomal pathway activity and increased synaptic contacts within the rostrum of the corpus callosum of mHFD-exposed male and female P30 offspring

Considering the unaffected oligodendrocytic cell lineage density, distribution, and maturation, we next investigated the possible involvement of another cell type –microglia– in the myelination and cytosolic myelin channel changes that we characterized in the corpus callosum upon exposure to mHFD. Previous work by Graf and colleagues revealed elevation of several inflammatory transcripts concomitant to the hypomyelination measured in the medial prefrontal cortex of P21 male offspring exposed to a mHFD (Graf et al., 2016). Moreover, a growing body of evidence reveals the role of microglia in the developmental process of myelination (Hagemeyer et al., 2017; Wlodarczyk et al., 2017; Li et al., 2019; Hughes and Appel, 2020; Nemes-Baran et al., 2020). We sought to investigate potential changes in microglial number and distribution (spacing index, nearest neighbor distance, cluster analysis), as well as morphology, ultrastructure, and functional interactions with their microenvironment. We also assessed infiltrating myeloid cells, which could be another potential contributor. Our observations were made in P30 offspring of both sexes, among the rostrum of the corpus callosum.

**Fig. 4:**
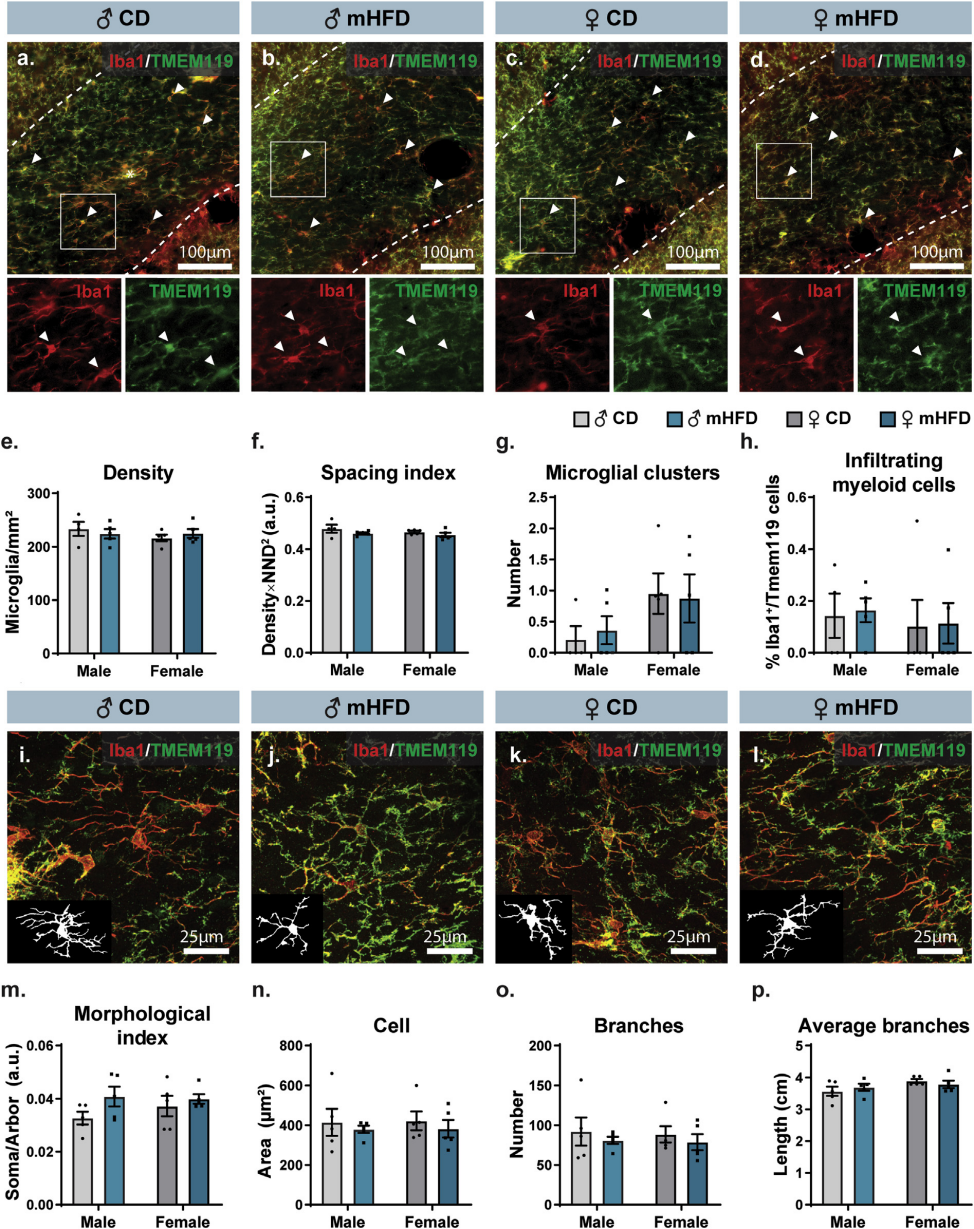
No significant changes in microglial density, distribution, and morphology in the rostrum of the corpus callosum of P30 offspring. (a–d) Representative pictures of Iba1 and TMEM119 staining illustrate the absence of difference between offspring microglial (e) density, (f) spacing, and (g) clustering, as well as (h) infiltrating myeloid cells (3–4 sections/animal, n ​= ​5 animals/group). On the pictures, the rostrum is delimitated by white dashed lines, while an asterisk (∗) indicates a cell cluster and white arrowheads indicate a few on-focus microglia within the region. Zoom-in insets of 100 ​μm ​× ​100 ​μm square of Iba1 (left side in red) and TMEM119 (right side in green) show the colocalization of the staining. (i–l) Representative pictures of microglia illustrate the similitude of microglial morphology between the offspring groups, notably regarding microglial (m) morphological index, (n) cell area, (o) number of branches, and (p) average length of branches (17–22 microglia/animal, n ​= ​5 animals/group). Bar graphs represent Mean ​± ​SEM. ♂: male, ♀: female, CD: control diet, mHFD: maternal high-fat diet, NND: nearest neighbor distance. (For interpretation of the references to color in this figure legend, the reader is referred to the Web version of this article.)

**Fig. 5:**
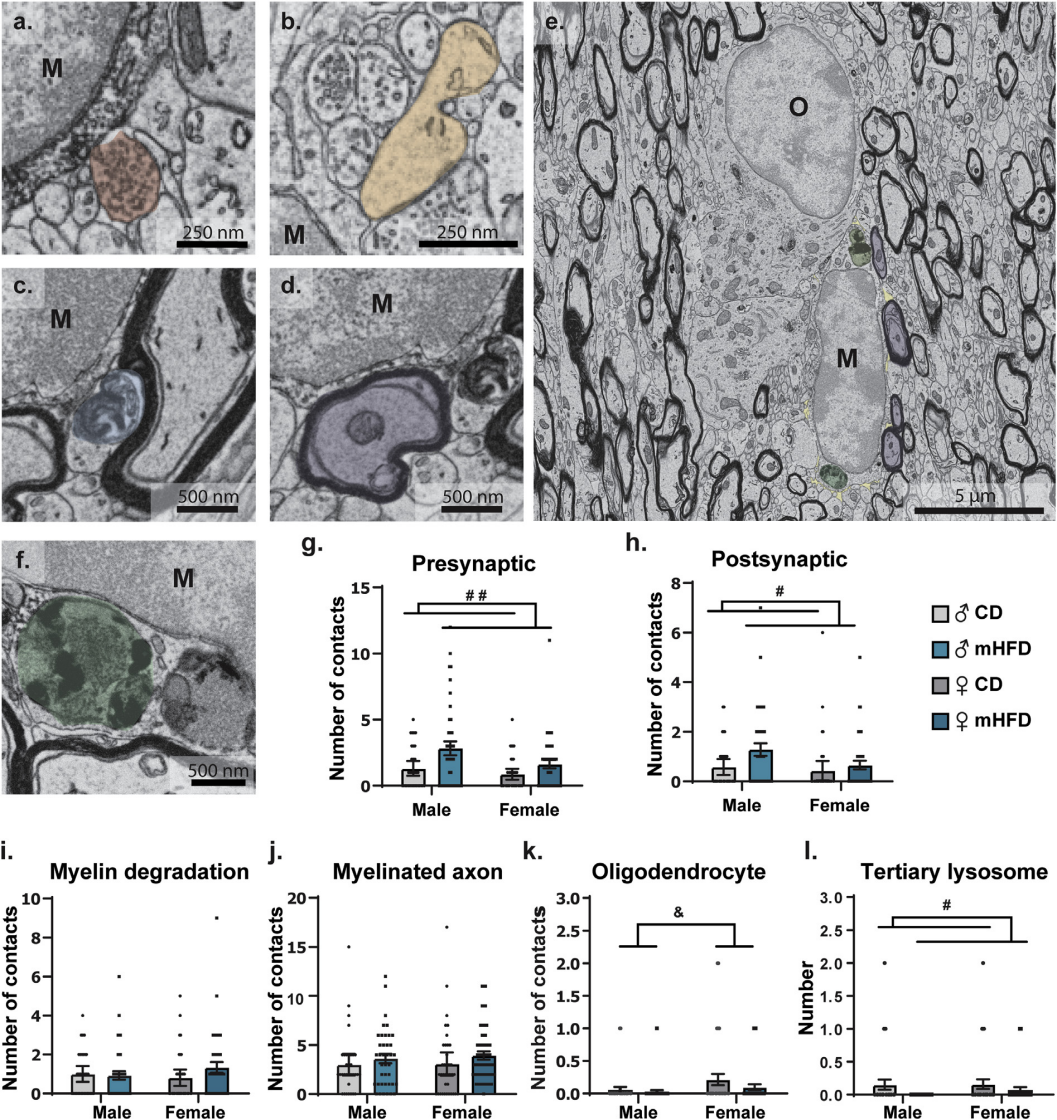
Microglia present increased interactions with synaptic elements and reduced tertiary lysosomes in P30 male and female offspring exposed to mHFD, while microglia-oligodendrocyte interactions are more frequent in female than male offspring. (a–f) Representative pictures show main ultrastructural parameters studied, including (a,g) interactions with presynaptic axon terminals, (b,h) postsynaptic dendritic spines, (c,i) degraded myelin, (d,j) myelinated axons, and (e,k) myelinating cells, as well as (g) number of tertiary lysosomes (n ​= ​33–41 microglia/group, N ​= ​4 animals/group). Organelles and microenvironment elements are pseudocolored in their respective representative pictures. Cell bodies are annotated with “M” for microglia or “O” for oligodendrocytes. Bar graphs represent Mean ​± ​SEM. Statistical significance was assessed by mixed effect model, where ^#^*p* ​< ​0.05, ^# #^*p* ​< ​0.05 for *Diet effect*, ^&^*p* ​< ​0.05 for *Sex effect*. ♂: male, ♀: female, CD: control diet, M: microglia, O: oligodendrocyte, mHFD: maternal high-fat diet.

Using immunofluorescence staining against Iba1 and TMEM119, we first characterized the density, distribution and clustering of microglia (Iba1^+^/TMEM119^+^) as well as the number of infiltrating myeloid cells (Iba1^+^/TMEM119^-^). For all of these parameters, no significant difference was observed between offspring groups (Fig. 4a–h, Supplementary Table 3). Assessment of microglial morphology also showed no difference between groups regarding microglial area (soma, arbor, cell), shape (morphological index, circularity, solidity, aspect ratio), and organization of their arborization (number of branches, average branch length, longest branch, number of junctions) (Fig. 4, Supplementary Table 4). At the ultrastructural level, upon exposure to mHFD, microglial cell bodies nevertheless interacted differently with their microenvironment, regardless of the sex of the offspring. We found that mHFD-exposed microglia exhibited augmented numbers of contacts with synaptic structures, pre- and postsynaptic, suggesting an increased modulation of synaptic activity, without changes in their relationships with myelinated axons as well as degraded myelin (Fig. 5a–d,g-j, Table 4). Moreover, mHFD-exposed microglia had reduced numbers of mature tertiary lysosomes, suggesting an impaired phagolysosomal pathway activity (Fig. 5f,l, Table 4). At P30, female *vs* male offspring, regardless of their maternal diet, showed an increased number of microglial cell bodies contacting oligodendrocyte cell bodies. This sex difference of microglia-oligodendrocyte interaction may lead to differential outcomes in male *vs* female offspring following microglial functional alterations, with consequences on myelination and the neuronal network, i.e., impaired phagolysosomal activity and increased synaptic modulation, after mHFD.

**Table 4:** mHFD effects on microglial ultrastructure in the rostrum of the corpus callosum of P30 offspring. n ​= ​33–41 microglia/group, N ​= ​4 animals/group. Statistical significance was assessed by mixed effect model, where ∗ *p* ​< ​0.05, ∗∗*p* ​< ​0.01. #: number, CD: control diet, ER/Golgi: endoplasmic reticulum and Golgi apparatus, mHFD: maternal high-fat diet.

Parameters			Mean ​± ​standard error of the mean				*F*	*p*
			Male		Female			
			CD	mHFD	CD	mHFD		
Organelles	# Lysosomes	Primary	1.727 ​± ​0.434	2.000 ​± ​0.447	1.892 ​± ​0.487	2.200 ​± ​0.476	Sex∗Diet: 0.003469 Sex: 0.1699 Diet: 0.4150	Sex∗Diet: 0.9531 Sex: 0.6808 Diet: 0.5205
		Secondary	0.273 ​± ​0.090	0.359 ​± ​0.101	0.459 ​± ​0.138	0.390 ​± ​0.120	Sex∗Diet: 0.4536 Sex: 0.8919 Diet: 0.005444	Sex∗Diet: 0.5017 Sex: 0.3465 Diet: 0.9413
		Tertiary	0.152 ​± ​0.077	0.000 ​± ​0.000	0.162 ​± ​0.073	0.073 ​± ​0.041	Sex∗Diet: 0.3312 Sex: 0.5953 Diet: 4.901	Sex∗Diet: 0.5658 Sex: 0.4416 Diet: **0.0284∗**
	# Lipidic inclusion		0.242 ​± ​0.107	0.128 ​± ​0.066	0.162 ​± ​0.073	0.122 ​± ​0.062	Sex∗Diet: 0.2340 Sex: 0.3197 Diet: 1.019	Sex∗Diet: 0.6293 Sex: 0.5726 Diet: 0.3145
	# Endosomes	Empty	0.182 ​± ​0.068	0.128 ​± ​0.066	0.189 ​± ​0.076	0.146 ​± ​0.066	Sex∗Diet: 0.006046 Sex: 0.03394 Diet: 0.4854	Sex∗Diet: 0.9381 Sex: 0.8541 Diet: 0.4871
		Content	0.455 ​± ​0.116	0.513 ​± ​0.151	0.378 ​± ​0.131	0.439 ​± ​0.171	Sex∗Diet: 6.439 ×10^-^^5^ Sex: 0.2576 Diet: 0.1620	Sex∗Diet: 0.9936 Sex: 0.6125 Diet: 0.6879
	# Dilated ER/golgi cisternae		6.939 ​± ​1.021	8.256 ​± ​1.190	7.189 ​± ​1.789	8.098 ​± ​1.906	Sex∗Diet: 0.01696 Sex: 0.0008399 Diet: 0.5029	Sex∗Diet: 0.8966 Sex: 0.9769 Diet: 0.4794
	# Mitochondria	Elongated	0.394 ​± ​0.150	0.462 ​± ​0.137	0.324 ​± ​0.140	0.732 ​± ​0.229	Sex∗Diet: 0.9589 Sex: 0.3341 Diet: 1.874	Sex∗Diet: 0.3291 Sex: 0.5642 Diet: 0.1731
		Total	2.697 ​± ​0.373	2.718 ​± ​0.442	3.162 ​± ​0.763	2.951 ​± ​0.549	Sex∗Diet: 0.04288 Sex: 0.3889 Diet: 0.02877	Sex∗Diet: 0.8362 Sex: 0.5338 Diet: 0.8655
Interactions with their microenvironment	# Synaptic elements	Pre	1.303 ​± ​0.273	2.821 ​± ​0.526	0.865 ​± ​0.202	1.634 ​± ​0.320	Sex∗Diet: 1.071 Sex: 5.051 Diet: 10.01	Sex∗Diet: 0.3023 Sex: **0.0261∗** Diet: **0.0019∗∗**
		Post	0.576 ​± ​0.157	1.282 ​± ​0.257	0.432 ​± ​0.192	0.659 ​± ​0.173	Sex∗Diet: 1.411 Sex: 3.598 Diet: 5.319	Sex∗Diet: 0.2368 Sex: 0.0598 Diet: **0.0225∗**
	# Myelinated axon		3.000 ​± ​0.538	3.615 ​± ​0.478	3.054 ​± ​0.586	3.927 ​± ​0.425	Sex∗Diet: 0.06436 Sex: 0.1298 Diet: 2.151	Sex∗Diet: 0.8001 Sex: 0.7192 Diet: 0.1446
	# Degraded myelin		1.000 ​± ​0.199	0.923 ​± ​0.215	0.811 ​± ​0.204	1.341 ​± ​0.272	Sex∗Diet: 1.745 Sex: 0.2483 Diet: 0.9732	Sex∗Diet: 0.1886 Sex: 0.6190 Diet: 0.3255
	# Contacts with brain cells	Astrocyte	0.061 ​± ​0.042	0.154 ​± ​0.059	0.243 ​± ​0.081	0.049 ​± ​0.034	Sex∗Diet: 6.371 Sex: 0.4631 Diet: 0.7886	Sex∗Diet: **0.0127∗** Sex: 0.4972 Diet: 0.3760
		Neuron	0.061 ​± ​0.042	0.051 ​± ​0.036	0.135 ​± ​0.069	0.024 ​± ​0.024	Sex∗Diet: 1.263 Sex: 0.2786 Diet: 1.770	Sex∗Diet: 0.2629 Sex: 0.5984 Diet: 0.1855
		Oligodendrocyte	0.061 ​± ​0.042	0.00.26 ​± ​0.026	0.216 ​± ​0.088	0.098 ​± ​0.047	Sex∗Diet: 0.5692 Sex: 4.207 Diet: 1.918	Sex∗Diet: 0.4518 Sex: **0.0420∗** Diet: 0.1682
		Oligodendrocyte progenitor	0.000 ​± ​0.000	0.026 ​± ​0.026	0.054 ​± ​0.038	0.024 ​± ​0.024	Sex∗Diet: 1.083 Sex: 0.9869 Diet: 0.005728	Sex∗Diet: 0.2998 Sex: 0.3221 Diet: 0.9398
		Blood vessel	0.091 ​± ​0.051	0.077 ​± ​0.043	0.027 ​± ​0.027	0.049 ​± ​0.034	Sex∗Diet: 0.2075 Sex: 1.376 Diet: 0.009800	Sex∗Diet: 0.6494 Sex: 0.2428 Diet: 0.9213
	# Extracellular space pocket		4.182 ​± ​0.512	3.590 ​± ​0.575	3.216 ​± ​0.384	3.610 ​± ​0.511	Sex∗Diet: 0.9501 Sex: 0.8745 Diet: 0.03855	Sex∗Diet: 0.3313 Sex: 0.3512 Diet: 0.8446
	# Extracellular digestion		0.455 ​± ​0.145	0.308 ​± ​0.098	0.216 ​± ​0.088	0.293 ​± ​0.112	Sex∗Diet: 1.009 Sex: 1.298 Diet: 0.1002	Sex∗Diet: 0.3169 Sex: 0.2565 Diet: 0.7521

### 3.4 Loss of social memory and altered sensorimotor gating in mHFD-exposed adolescent male and female offspring

**Fig. 6:**
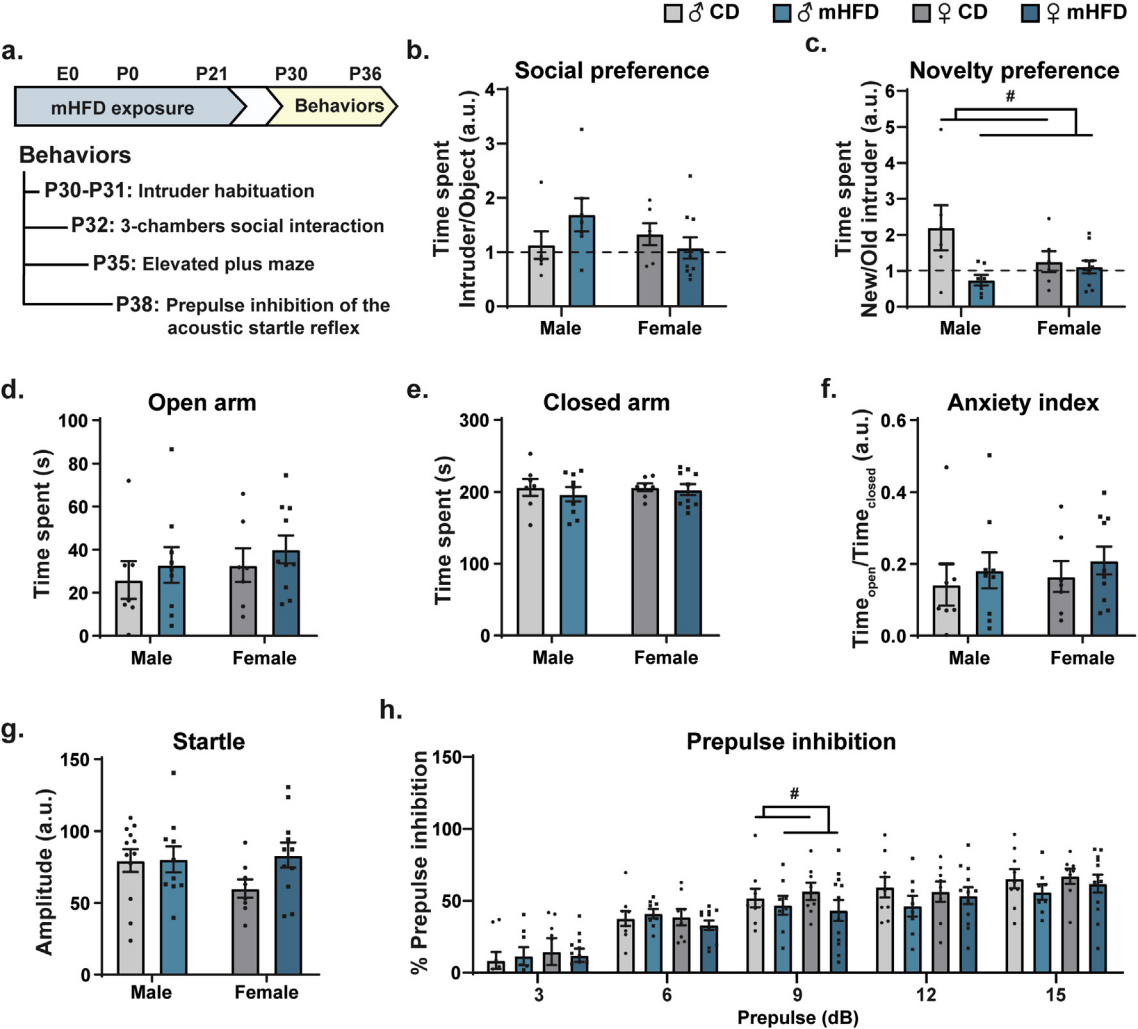
mHFD leads to loss of social novelty preference and reduces prepulse inhibition of the acoustic reflex in adolescent male and female adolescent offspring. (a) Timeline of behavioral assessment is illustrated. (b–c) Using three-chambers social interaction test, (b) social preference, and (c) social novelty preference were evaluated (n ​= ​6–8 litters/group, N ​= ​6–11 animals/group). (d–f) Anxiety-related behaviors were assessed using elevated plus maze test and by measuring time spent in (d) open arms and (e) closed arms, as well as (f) anxiety index (n ​= ​6–8 litters/group, N ​= ​6–11 animals/group). Finally, sensorimotor gating was characterized during prepulse inhibition of the startle acoustic reflex, looking at both (g) startle response and (h) inhibition of the startle response after exposure to a prepulse ranging from 3 ​dB to 15 ​dB (n ​= ​7–10 litters/group, N ​= ​8–12 animals/group). Bar graphs represent Mean ​± ​SEM. Statistical significance was assessed using a mixed effect model for social and anxiety-related behaviors, and by two-way ANOVA for sensorimotor gating, where ^#^*p* ​< ​0.05 for *Diet effect*. ♂: male, ♀: female, a.u. arbitrary unit, CD: control diet, mHFD: maternal high-fat diet, P: postnatal day.

Myelination is crucial for an efficient neuronal signal transduction during brain communication and its alteration has been linked to several neurodevelopmental disorders (Fields, 2008; Drakesmith et al., 2016; Hardan et al., 2016; Watanabe et al., 2018). We assessed in the adolescent offspring exposed to mHFD whether the changes in myelin organization and myelin-microglial interactions observed could lead to behavioral alterations (Fig. 6a) associated in human with neurodevelopmental disorders (Leonardo and Hen, 2008; Mena et al., 2016; Isaksson et al., 2019). Considering the short timeframe of adolescence in mouse, we focused on social, anxiety-related and sensorimotor gating behaviors, which notably require proper bidirectional communication between the prefrontal cortex and the hippocampus (Tanimizu et al., 2017; Sun et al., 2020; Shin and Liberzon, 2010; Tapias-Espinosa et al., 2019; Miller et al., 2010).

Evaluation of social behaviors revealed similar social preference between CD- and mHFD-exposed offspring (Fig. 6b, Supplementary Table 5), whereas social novelty preference was decreased in mHFD-exposed offspring compared to CD-exposed offspring, regardless of their sex (Fig. 6c, Supplementary Table 5). During the elevated plus maze task, no change in time spent within the open or closed arms, nor in the anxiety index, was found after mHFD exposure (Fig. 6d–f, Supplementary Table 5). In the prepulse inhibition of the acoustic startle reflex test, offspring showed similar startle responses (Fig. 6g, Supplementary Table 5), but mHFD-exposed offspring had reduced prepulse inhibition specifically with the 9 ​dB prepulse compared to control groups, regardless of their sex (Fig. 6h, Supplementary Table 5).

## 4 Discussion

**Fig. 7:**
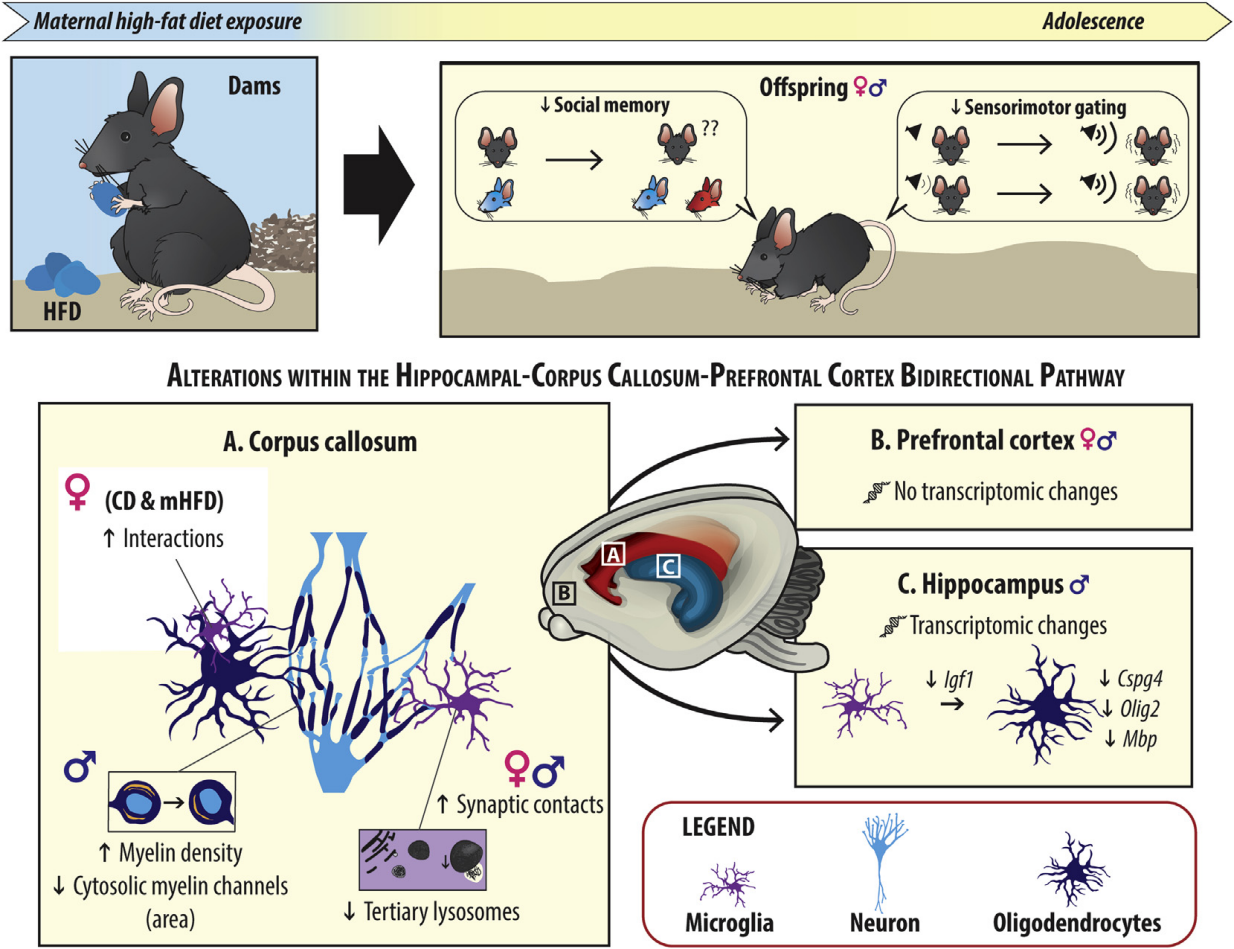
mHFD leads to myelination changes within the hippocampal-corpus callosum-prefrontal cortex bidirectional pathway of P30 mouse adolescent offspring associated with behavioral changes. ♂: male, ♀: female, ↓: lower, ↑: higher, CD: control diet, *Cspg4*: chondroitin sulfate proteoglycan 4, HFD: high-fat diet, *Igf1*: insulin-like growth factor 1, *Mbp*: myelin basic protein, mHFD: maternal high-fat diet, *Olig2*: oligodendrocyte factor 2.

In the present study, we found that mHFD induced sex-specific alterations in myelin organization and microglial interactions with their microenvironment in P30 male offspring. Predominant findings are a reduced number and area of myelin cytosolic channels in the rostrum of the corpus callosum. We also found decreased myelination-associated transcripts and myelin-promoting growth factors, known to be mainly expressed by microglia, in the hippocampus −a key projection region of the corpus callosum. In the corpus callosum, the reduced number and area of myelin cytosolic channels were not accompanied by differences in the density, distribution or maturation of oligodendrocytic cell populations but were concomitant with changes in microglial phagolysosomal pathway and ultrastructural interactions with synaptic elements. Parallel to these changes in the adolescent brain, male and female offspring exposed to a mHFD showed altered social novelty preference and a reduced capacity to inhibit the acoustic startle reflex during a prepulse inhibition test for sensorimotor gating (Fig. 7).

Our results in mHFD-exposed adolescent male offspring highlight a reduced number and area of cytosolic myelin channels in the corpus callosum. Cytosolic channels, also known as myelinic or cytoplasmic channels, are an important component of the axon-myelin unit that has been hypothesized to contribute to efficient neuronal communication, action potential propagation, and axonal metabolism by providing adequate energy substrates (Snaidero et al., 2017; Stassart et al., 2018). Loss of cytosolic myelin channels was previously shown to result from a disequilibrium in myelin protein content caused by genetic deletion of CNP (2′,3′-cyclic nucleotide 3′-phosphodiesterase), which was rescued by knocking-out MBP in mice (Snaidero et al., 2014, 2017). CNP inhibits premature compaction of myelin (Snaidero et al., 2017) and its expression by mature myelinating oligodendrocytes plays a crucial role in stabilizing and maintaining the myelin structure (Domingues et al., 2018). However, in our study, the oligodendrocytic maturation rate remained similar between offspring groups, suggesting that a change in oligodendrocyte-released CNP was unlikely to mediate the effects we observed upon exposure to mHFD.

Indeed, further characterization of our model revealed that the reduced myelinic channels in mHFD-exposed male offspring were not accompanied by a change in oligodendrocyte density, distribution or maturation. In mice exposed to HFD during adulthood, a loss of NG2^+^ oligodendrocyte progenitors, Olig2^+^ oligodendrocytes and CC-1^+^ myelinating oligodendrocytes (associated with mitochondrial dysfunction and oxidative stress) was observed across the central nervous system (Langley et al., 2020). In mice exposed to mHFD *in utero*, Graf and colleagues reported a reduction of Olig2^+^ cell number in the lateral cortex as well as reduced MBP-immunoreactive areas in the medial cortex of P7 and P21 offspring (Graf et al., 2016), which could suggest a reduction of oligodendrocyte populations including myelinating ones. Contrary to these results, we did not observe a significant change in the expression of genes associated with myelination processes in the prefrontal cortex. This discrepancy could be explained either by differences in the time course of the myelination changes that may be normalized in the prefrontal cortex by P30, or a difference between the approaches used (mRNA *vs* protein expression). Nevertheless, in the hippocampus, we did identify reduced expression of the myelination-associated genes *Mbp*, *Olig2* and *Cspg4*, as well as of a myelinating factor mainly expressed by microglia, *Igf1* (Hagemeyer et al., 2017; Wlodarczyk et al., 2017; Li et al., 2019). In mice, both the corpus callosum and the hippocampus reach fiber maturation around the same developmental stage (between P30-40) –the age of interest in the current study, while cerebral cortex myelination reaches steady-state at P10 (Baloch et al., 2009). The differential expression we found between the prefrontal cortex and hippocampus may be due to this regional variation in the developmental time-course of myelination, while sex-specific transcriptomic changes observed could be either driven by inflammation or epigenetic modulation induced by mHFD (Bordeleau et al., 2021).

Instead of a shift in the oligodendrocytic cell population, our results revealed a potential involvement of microglia, which increased their synaptic contacts and presented reduced numbers of tertiary lysosomes among the corpus callosum upon mHFD. Constitutive lysosomal alterations were previously associated with myelination anomalies (Renaud, 2012; Nave and Werner, 2014; Shen et al., 2016b). During myelination, the phagolysosomal pathway that involves lysosomes is critical for myelin turnover and compaction (Shen et al., 2016b). It has been recently shown that myelin proteins, like PLP, can localize to late endosomes/lysosomes and be transported to the plasma membrane for extracellular release (Shen et al., 2016b). Exosomes containing PLP can be further taken up by microglia, then degraded *via* the phagolysosomal pathway (Fitzner et al., 2011). Besides extracellular myelin protein regulation, microglial release of proteinases is important for modulating the extracellular matrix composition and geometry, which can influence myelin compaction (Domingues et al., 2018). Moreover, the increased interactions of microglia with synapses can influence myelination processes *via* activity-dependent myelination (Fields, 2015), occurring through the axon-myelin pathway that implicates cytosolic myelin channels (Micu et al., 2016; Hughes and Appel, 2019). In microglia, functional lysosomes are needed for efficient progression of phagocytosis and autophagy (Shen et al., 2016a). Disruption of microglial pruning of myelin sheaths in zebrafish caused excessive and ectopic myelin during development (Hughes and Appel, 2020). Insufficient or reduced phagocytic activity by microglia could lead to similar excessive and/or ectopic myelin, resulting in increased myelin density as reported in the current study. Moreover, previous work by our team has revealed reduced transcript levels of microglial receptors known to mediate their interactions with myelin, including *Trem2* (Takahashi et al., 2007; Cignarella et al., 2020) and *Cx3cr1* (Lampron et al., 2015), in male offspring exposed to mHFD (Bordeleau et al., 2020). Together, these findings point to a potential microglial implication in the observed myelin alterations. Moreover, the microglial receptors Cx3cr1 and Trem2 have been shown to mediate their phagocytosis and synaptic remodeling processes (Thrash et al., 2009; Noda et al., 2011; Paolicelli et al., 2011; Chertoff et al., 2013; Zhan et al., 2014; Kawabori et al., 2015; Filipello et al., 2018), which by modulating neuronal activity can further influence myelination (Fields, 2015). Alongside changes of these microglial receptors, we have previously reported in our mHFD model reduced mRNA levels of the inflammatory-mediating cytokine *Tgf1b* (Bordeleau et al., 2020), which has been shown to modulate the motility of primary mouse oligodendrocytes *in vitro* (Lalive et al., 2005)*.*

It remains possible that the myelin changes reported in the current study also involve other brain cells, like astrocytes (Domingues et al., 2016), or other mechanisms unexplored due to limitations of our experimental design, such as changes in the release of CNP by oligodendrocytes and microglia (Wu et al., 2006; Yang et al., 2014) or changes in the properties of structural myelin proteins (e.g., CNP enzymatic activities, myelin composition, and overall charge modulating adhesive characteristics) (Chang et al., 2016; Domingues et al., 2018).

Our current observations suggest that mHFD-induced alterations along the hippocampus-corpus callosum-prefrontal cortex pathway are associated with social memory and sensorimotor gating deficits in male and female adolescent mouse offspring. Considering that the behavioral outcomes were similar in both sexes, mHFD may possess a broader impact on the offspring, where developmental trajectories (Andersen, 2003; Lenroot et al., 2007) and microglial cell functions (Bordeleau et al., 2019, Lenz et al., 2011, Lenz et al., 2013, Villa et al., 2018) may diverge between sexes. Alternatively, microglia –key cells in several neurodevelopmental processes including myelination– are also known to be broadly impacted by mHFD at metabolic and inflammatory levels (Bilbo and Schwarz, 2009; Bilbo and Tsang, 2010; Bordeleau et al., 2021). In regard to the latter, future studies directly modulating microglial capacity to respond to mHFD-driven inflammation (e.g., conditional knock out, pharmacological treatment in the mother) could unravel the interplay between microglia, myelin changes and the behavioral outcomes reported.

## 5 Conclusions

Our results provide novel insights into mHFD-induced myelin changes during adolescence in a mouse model and highlight the importance of studying microglia-mediated processes. Although previous papers have hinted at myelination changes, our study used for the first time a nanoscale super-resolution approach, and reports evidence of changes in myelin structural organization and microglial direct interactions. Importantly, we discovered that mHFD led to a reduction of cytosolic myelin channels in the males specifically. While investigating one of the most important white matter regions of the brain: the corpus callosum, we surprisingly found via our combined approach of cellular and ultrastructural techniques that myelin changes were not attributed to the oligodendrocytes but associated with changes in the functional interactions of microglia within the corpus callosum. Therefore, the impact of mHFD on developmental myelination might contribute to the discrepancies between sexes often observed in neurodevelopmental disorders such as autism spectrum disorders and schizophrenia.

## Data sharing

Original data supporting the findings of this study are available from the corresponding authors upon reasonable request.

## Declaration of competing interest

The authors disclose no competing financial interests.
