# Supplementary material for: Maternal high-fat diet modifies myelin organization, microglial interactions, and results in social memory and sensorimotor gating deficits in adolescent mouse offspring
Source: Brain Behav Immun Health. 2021 Jun 9;15:100281. doi: 10.1016/j.bbih.2021.100281 (PMC8474164; doi:10.1016/j.bbih.2021.100281)
Supplement: Multimedia component 1 [file mmc1.docx]

## Supplementary Material

**Supplementary Table 1. mHFD effects on area and optical density of the corpus callosum of P30 offspring measured by Sudan Black B staining.** a.u.: arbitrary unit, CD: control diet, mHFD: maternal high-fat diet.

| **Parameters** | | **Mean ± standard error of the mean** | | | | ***F*** | ***p*** |
| --- | --- | --- | --- | --- | --- | --- | --- |
|  |  | **Male** | | **Female** | |  |  |
|  |  | **CD** | **mHFD** | **CD** | **mHFD** |  |  |
| Area (mm²) | Rostrum | 1.867 ±0.047 | 2.034 ±0.129 | 2.057 ±0.083 | 1.876 ±0.085 | Sex*Diet: 3.665  Sex: 0.02982  Diet: 0.005266 | Sex*Diet: 0.0736  Sex: 0.8651  Diet: 0.9431 |
|  | Genu | 1.964 ±0.066 | 2.046 ±0.091 | 2.136 ±0.036 | 1.897 ±0.049 | Sex*Diet: 6.304  Sex: 0.03415  Diet: 1.501 | Sex*Diet: **0.0232***  Sex: 0.8557  Diet: 0.2382 |
|  | Body | 1.580 ±0.041 | 1.621 ±0.047 | 1.732 ±0.066 | 1.574 ±0.037 | Sex*Diet: 4.124  Sex: 1.148  Diet: 1.408 | Sex*Diet: 0.0593  Sex: 0.2998  Diet: 0.2527 |
|  | Splenium | 1.399 ±0.049 | 1.478 ±0.065 | 1.457 ±0.171 | 1.341 ±0.109 | Sex*Diet: 0.7989  Sex: 0.1314  Diet:0.02854 | Sex*Diet: 0.3847  Sex: 0.7217  Diet: 0.8680 |
|  | Average of whole corpus callosum | 1.702 ±0.034 | 1.795 ±0.048 | 1.845 ±0.057 | 1.672 ±0.065 | Sex*Diet: 6.506  Sex: 0.8483  Diet: 0.4493 | Sex*Diet: **0.0214***  Sex: 0.8483  Diet: 0.4493 |
| Optical density (a.u.) | Rostrum | 0.192 ±0.018 | 0.262 ±0.012 | 0.259 ±0.007 | 0.258 ±0.013 | Sex*Diet: 7.241  Sex: 5.918  Diet: 7.092 | Sex*Diet: **0.0161***  Sex: **0.0271***  Diet: **0.0170*** |
|  | Genu | 0.276 ±0.020 | 0.294 ±0.020 | 0.292 ±0.008 | 0.273 ±0.007 | Sex*Diet: 1.460  Sex: 0.03312  Diet: 0.001066 | Sex*Diet: 0.2446  Sex: 0.8579  Diet: 0.9744 |
|  | Body | 0.253 ±0.014 | 0.303 ±0.014 | 0.319 ±0.005 | 0.305 ±0.010 | Sex*Diet: 7.273  Sex: 8.417  Diet: 2.340 | Sex*Diet: **0.0159***  Sex: **0.0104***  Diet: 0.1456 |
|  | Splenium | 0.250 ±0.008 | 0.299 ±0.011 | 0.316 ±0.016 | 0.320 ±0.012 | Sex*Diet: 3.396  Sex: 12.83  Diet: 4.498 | Sex*Diet: 0.0840  Sex: **0.0025****  Diet: **0.0499*** |
|  | Average of whole corpus callosum | 0.241 ±0.014 | 0.288 ±0.005 | 0.295 ±0.005 | 0.288 ±0.008 | Sex*Diet: 10.03  Sex: 10.00  Diet: 5.332 | Sex*Diet: **0.0060****  Sex: **0.0060****  Diet: **0.0346*** |

**Supplementary Table 2. mHFD effects on transcript levels in the prefrontal cortex and the hippocampus of P30 male and female offspring.** 2^-ΔΔCt^: relative expression, ΔΔCt: fold change of expression, CD: control diet, *Cspg4*: chondroitin sulfate proteoglycan 4, *Gpnmb*: glycoprotein Nmb, *Igf1*: insulin-like growth factor 1, *Mbp*: myelin basic protein, mHFD: maternal high-fat diet, *Olig2*: oligodendrocyte factor 2, *Plp1*: proteolipid protein 1, *Rpl32*: ribosomal protein L32.

| **Gene** | **Male** | | **Female** | | ***F*** | ***p*** |
| --- | --- | --- | --- | --- | --- | --- |
|  | **CD** | **mHFD** | **CD** | **mHFD** |  |  |
| **Prefrontal Cortex** | | | | | | |
| ***Mbp*** | 2^-ΔΔCt^= 1.000  ΔΔCt= 0.000  ± 0.171 | 2^-ΔΔCt^= 0.996  ΔΔCt= 0.006  ± 0.285 | 2^-ΔΔCt^= 0.836  ΔΔCt= 0.258  ± 0.367 | 2^-ΔΔCt^= 0.893  ΔΔCt=0.163  ± 0.351 | Sex*Diet: 0.03202  Sex: 0.4449  Diet: 0.02144 | Sex*Diet: 0.8599  Sex: 0.5128  Diet: 0.8851 |
| ***Plp1*** | 2^-ΔΔCt^= 1.000  ΔΔCt= 0.000  ± 0.195 | 2^-ΔΔCt^= 0.779  ΔΔCt= 0.360  ± 0.313 | 2^-ΔΔCt^= 0.766  ΔΔCt= 0.385  ± 0.357 | 2^-ΔΔCt^= 0.776  ΔΔCt= 0.366  ± 0.361 | Sex*Diet: 0.3431  Sex: 0.3812  Diet: 0.3070 | Sex*Diet: 0.5649  Sex: 0.5443  Diet: 0.5860 |
| ***Cspg4*** | 2^-ΔΔCt^= 1.000  ΔΔCt= 0.000  ± 0.189 | 2^-ΔΔCt^= 0.932  ΔΔCt= 0.101  ± 0.194 | 2^-ΔΔCt^=1.258  ΔΔCt= -0.332  ± 0.254 | 2^-ΔΔCt^=0.950  ΔΔCt= 0.074  ± 0.323 | Sex*Diet: 0.3545  Sex: 0.5105  Diet: 0.9848 | Sex*Diet: 0.5586  Sex: 0.4836  Diet: 0.3335 |
| ***Olig2*** | 2^-ΔΔCt^= 1.000  ΔΔCt= 0.000  ± 0.131 | 2^-ΔΔCt^= 0.914  ΔΔCt= 0.130  ± 0.189 | 2^-ΔΔCt^= 1.124  ΔΔCt= -0.169  ± 0.244 | 2^-ΔΔCt^= 0.958  ΔΔCt= 0.062  ± 0.280 | Sex*Diet: 0.05054  Sex: 0.2911  Diet: 0.6550 | Sex*Diet: 0.8245  Sex: 0.5958  Diet: 0.4283 |
| ***Igf1*** | 2^-ΔΔCt^= 1.000  ΔΔCt= 0.000  ±0.322 | 2^-ΔΔCt^= 1.040  ΔΔCt= -0.056  ± 0.259 | 2^-ΔΔCt^= 0.747  ΔΔCt= 0.421  ± 0.271 | 2^-ΔΔCt^= 0.843  ΔΔCt= 0.246  ± 0.419 | Sex*Diet: 0.02755  Sex: 1.213  Diet: 0.1204 | Sex*Diet: 0.8699  Sex: 0.2845  Diet: 0.7324 |
| ***Gpnmb*** | 2^-ΔΔCt^= 1.000  ΔΔCt= 0.000  ± 0.525 | 2^-ΔΔCt^= 1.119  ΔΔCt= -0.162  ± 0.243 | 2^-ΔΔCt^= 0.912  ΔΔCt= 0.134  ± 0.329 | 2^-ΔΔCt^= 0.896  ΔΔCt= 0.158  ± 0.512 | Sex*Diet: 0.0476  Sex: 0.2675  Diet: 0.02232 | Sex*Diet: 0.8295  Sex: 0.6110  Diet: 0.8828 |
| **Hippocampus** | | | | | | |
| ***Mbp*** | 2^-ΔΔCt^= 1.000  ΔΔCt= 0.000  ± 0.287 | 2^-ΔΔCt^= 0.257  ΔΔCt= 1.959  ± 0.364 | 2^-ΔΔCt^= 0.964  ΔΔCt= 0.054  ± 0.314 | 2^-ΔΔCt^= 1.146  ΔΔCt= -0.197  ± 0.236 | Sex*Diet: 13.58  Sex: 12.38  Diet: 8.130 | Sex*Diet: **0.0016****  Sex: **0.0023****  Diet: **0.0102*** |
| ***Plp1*** | 2^-ΔΔCt^= 1.000  ΔΔCt= 0.000  ± 0.316 | 2^-ΔΔCt^= 0.541  ΔΔCt= 0.886  ± 0.422 | 2^-ΔΔCt^= 0.882  ΔΔCt= 0.181  ± 0.364 | 2^-ΔΔCt^= 1.060  ΔΔCt= -0.084  ± 0.258 | Sex*Diet: 2.867  Sex: 1.353  Diet: 0.8604 | Sex*Diet: 0.1068  Sex: 0.2592  Diet: 0.3653 |
| ***Cspg4*** | 2^-ΔΔCt^= 1.000  ΔΔCt= 0.000  ± 0.266 | 2^-ΔΔCt^= 0.106  ΔΔCt= 3.238  ± 0.290 | 2^-ΔΔCt^= 0.974  ΔΔCt= 0.038  ± 0.216 | 2^-ΔΔCt^= 1.058  ΔΔCt= -0.081  ± 0.235 | Sex*Diet: 44.56  Sex: 42.46  Diet: 38.42 | Sex*Diet: **<0.0001******  Sex: **<0.0001******  Diet: **<0.0001****** |
| ***Olig2*** | 2^-ΔΔCt^= 1.000  ΔΔCt= 0.000  ± 0.303 | 2^-ΔΔCt^= 0.156  ΔΔCt= 2.677  ± 0.334 | 2^-ΔΔCt^= 0.884  ΔΔCt= 0.178  ± 0.195 | 2^-ΔΔCt^= 1.015  ΔΔCt= -0.022  ± 0.225 | Sex*Diet: 29.50  Sex: 22.58  Diet: 21.87 | Sex*Diet: **<0.0001******  Sex: **0.0001*****  Diet: **0.0002***** |
| ***Igf1*** | 2^-ΔΔCt^= 1.000  ΔΔCt= 0.000  ± 0.300 | 2^-ΔΔCt^= 0.372  ΔΔCt= 1.427  ± 0.428 | 2^-ΔΔCt^= 1.145  ΔΔCt= -0.195  ± 0.343 | 2^-ΔΔCt^= 1.268  ΔΔCt= -0.342  ± 0.234 | Sex*Diet: 5.801  Sex: 9.133  Diet: 3.916 | Sex*Diet: **0.0263***  Sex: **0.0070****  Diet: 0.0625 |
| ***Gpnmb*** | 2^-ΔΔCt^= 1.000  ΔΔCt= 0.000  ± 0.306 | 2^-ΔΔCt^= 0.464  ΔΔCt= 1.109  ± 0.514 | 2^-ΔΔCt^= 1.497  ΔΔCt= -0.582  ± 0.459 | 2^-ΔΔCt^= 1.305  ΔΔCt= -0.384  ± 0.234 | Sex*Diet:1.400  Sex: 7.243  Diet: 2.901 | Sex*Diet: 0.2514  Sex: **0.0145***  Diet: 0.1048 |

**Supplementary Table 3. mHFD effects on microglial density, distribution, and peripheral myeloid cell infiltration in the rostrum of the corpus callosum of P30 offspring.** % Infiltration: Average percentage of IBA1^+^/TMEM119^-^ cells on total myeloid cells count, a.u.: arbitrary unit, CD: control diet, mHFD: maternal high-fat diet.

| **Parameters** | **Mean ± standard error of the mean** | | | | ***F*** | ***p*** |
| --- | --- | --- | --- | --- | --- | --- |
|  | **Male** | | **Female** | |  |  |
|  | **CD** | **mHFD** | **CD** | **mHFD** |  |  |
| Density (cells/mm²) | 233.6 ±13.1 | 224.3 ±8.7 | 216.4 ±6.2 | 224.9 ±8.3 | Sex*Diet: 0.9794  Sex: 0.8556  Diet: 0.002384 | Sex*Diet: 0.3380  Sex: 0.3696  Diet: 0.9617 |
| Spacing index (a.u.) | 0.479 ±0.016 | 0.461 ±0.004 | 0.466 ±0.004 | 0.455 ±0.008 | Sex*Diet: 0.1543  Sex: 1.174  Diet: 3.191 | Sex*Diet: 0.7000  Sex: 0.2957  Diet: 0.0943 |
| Nearest neighbors distance (µm) | 45.55 ±0.97 | 45.52 ±0.86 | 46.60 ±0.53 | 45.20 ±1.11 | Sex*Diet: 0.5878  Sex: 0.1632  Diet: 0.6494 | Sex*Diet: 0.4552  Sex: 0.6919  Diet: 0.4329 |
| Cluster | 0.215 ±0.215 | 0.363 ±0.225 | 0.951 ±0.325 | 0.875 ±0.388 | Sex*Diet: 0.1336  Sex: 4.118  Diet: 0.01339 | Sex*Diet: 0.7198  Sex: 0.0606  Diet:0.9094 |
| % Infiltration | 0.143 ±0.085 | 0.164 ±0.046 | 0.102 ±0.102 | 0.114 ±0.078 | Sex*Diet: 0.003076  Sex: 0.3223  Diet: 0.04340 | Sex*Diet: 0.9565  Sex: 0.5786  Diet: 0.8378 |

**Supplementary Table 4. mHFD effects on morphological parameters of microglia in the rostrum of the corpus callosum of P30 offspring.** #: number, a.u.: arbitrary unit, CD: control diet, mHFD: maternal high-fat diet.

| **Parameters** | **Mean ± standard error of the mean** | | | | ***F*** | ***p*** |
| --- | --- | --- | --- | --- | --- | --- |
|  | **Male** | | **Female** | |  |  |
|  | **CD** | **mHFD** | **CD** | **mHFD** |  |  |
| Soma area (µm²) | 48.19 ±2.30 | 51.32 ±1.23 | 48.56 ±1.55 | 50.12 ±1.09 | Sex*Diet: 0.2379  Sex: 0.06582  Diet: 2.118 | Sex*Diet: 0.6323  Sex: 0.8008  Diet: 0.1649 |
| Arbor area (µm²) | 1366 ±182 | 1296  ±63 | 1522 ±135 | 1272 ±123 | Sex*Diet: 0.4528  Sex: 0.2467  Diet: 1.452 | Sex*Diet: 0.5106  Sex: 0.6262  Diet: 0.2457 |
| Morphological index (a.u.) | 0.033 ±0.002 | 0.041 ±0.004 | 0.037 ±0.004 | 0.040 ±0.002 | Sex*Diet: 0.7812  Sex: 0.3514  Diet: 0.3109 | Sex*Diet: 0.3899  Sex: 0.5616  Diet: 0.0969 |
| Cell area (µm²) | 414.7  ±68 | 379.7 ±18.2 | 421.6 ±46.9 | 382.2 ±44.0 | Sex*Diet: 0.002110  Sex: 0.009609  Diet: 0.6088 | Sex*Diet: 0.9639  Sex: 0.9231  Diet: 0.4466 |
| Circularity (a.u.) | 0.031 ±0.003 | 0.030 ±0.002 | 0.027 ±0.002 | 0.032 ±0.004 | Sex*Diet: 1.158  Sex: 0.2126  Diet:0.7533 | Sex*Diet: 0.2979  Sex: 0.6509  Diet: 0.3983 |
| Solidity (a.u.) | 0.288 ±0.008 | 0.277 ±0.006 | 0.263 ±0.010 | 0.285 ±0.010 | Sex*Diet: 3.575  Sex: 0.9591  Diet: 0.3689 | Sex*Diet: 0.0769  Sex: 0.3420  Diet: 0.5521 |
| Aspect ratio (a.u.) | 1.901 ±0.094 | 1.930 ±0.101 | 2.031 ±0.122 | 2.057 ±0.108 | Sex*Diet: 0.0002461  Sex: 1.436  Diet: 0.06491 | Sex*Diet: 0.9877  Sex: 0.2483  Diet: 0.8021 |
| # Branches | 92.05 ±17.66 | 80.95 ±4.66 | 88.51 ±10.37 | 78.75 ±10.12 | Sex*Diet: 0.003337  Sex: 0.06066  Diet: 0.8014 | Sex*Diet: 0.9547  Sex: 0.8086  Diet: 0.3839 |
| Average branch length (µm) | 3.566 ±0.146 | 3.689 ±0.109 | 3.889 ±0.060 | 3.782 ±0.117 | Sex*Diet: 1.048  Sex: 3.433  Diet:0.004982 | Sex*Diet: 0.3212  Sex: 0.0824  Diet: 0.9446 |
| Longest branch (µm) | 15.15 ±1.09 | 14.92 ±0.57 | 16.74 ±0.87 | 15.58 ±0.67 | Sex*Diet: 0.3181  Sex: 1.861  Diet: 0.7052 | Sex*Diet: 0.5806  Sex: 0.1914  Diet: 0.4134 |
| # Junctions | 47.22 ±9.40 | 41.24 ±2.55 | 45.47 ±5.69 | 40.26 ±5.45 | Sex*Diet: 0.003803  Sex: 0.04719  Diet: 0.7988 | Sex*Diet: 0.9516  Sex: 0.8308  Diet: 0.3847 |

**Supplementary Table 5. mHFD effects on social behaviors, anxiety-related behaviors and sensorimotor gating during adolescence.** a.u.: arbitrary unit, CD: control diet, mHFD: maternal high-fat diet.

| **Parameters** | | **Mean ± standard error of the mean** | | | | ***F*** | ***p*** |
| --- | --- | --- | --- | --- | --- | --- | --- |
|  |  | **Male** | | **Female** | |  |  |
|  |  | **CD** | **mHFD** | **CD** | **mHFD** |  |  |
| **Social behaviors** | | | | | | | |
| Social preference index (a.u.) | | 1.129 ±0.256 | 1.687 ±0.305 | 1.330 ±0.204 | 1.075 ±0.195 | Sex*Diet: 2.725  Sex: 0.6934  Diet: 0.3770 | Sex*Diet: 0.113  Sex: 0.4129  Diet: 0.5447 |
| Social novelty preference index (a.u.) | | 2.199 ±0.625 | 0.739 ±0.148 | 1.250 ±0.290 | 1.109 ±0.176 | Sex*Diet: 4.205  Sex: 0.8137  Diet: 6.199 | Sex*Diet: 0.0509  Sex: 0.3756  Diet: **0.0198*** |
| **Anxiety-related behaviors** | | | | | | | |
| Time spent in open arms (s) | | 26.016 ±8.778 | 32.966 ±8.315 | 32.826 ±7.727 | 40.120 ±6.469 | Sex*Diet: 0.0004780  Sex: 0.7856  Diet: 0.8174 | Sex*Diet: 0.9827  Sex: 0.3827  Diet: 0.3734 |
| Time spent in closed arms (s) | | 206.206 ±11.835 | 196.800 ±9.858 | 206.361 ±5.336 | 203.052 ±7.871 | Sex*Diet: 0.1104  Sex: 0.1220  Diet: 0.4802 | Sex*Diet: 0.7421  Sex: 0.7294  Diet: 0.4938 |
| Anxiety index (a.u.) | | 0.142 ±0.058 | 0.182 ±0.050 | 0.165 ±0.043 | 0.209 ±0.039 | Sex*Diet: 0.002224  Sex: 0.2756  Diet: 0.7800 | Sex*Diet: 0.9627  Sex: 0.6036  Diet: 0.3844 |
| **Sensorimotor gating** | | | | | | | |
| Startle response (a.u.) | | 79.517 ±7.977 | 80.350 ±8.975 | 60.063 ±6.416 | 83.236 ±8.743 | Sex*Diet: 1.755  Sex: 0.9650  Diet: 2.026 | Sex*Diet: 0.1934  Sex: 0.3323  Diet: 0.1630 |
| % Prepulse inhibition | 3dB | 14.079 ±5.521 | 7.559 ±3.814 | 14.628 ±9.361 | 11.676 ±4.996 | Sex*Diet: 0.09082  Sex: 0.1553  Diet: 0.6401 | Sex*Diet: 0.7648  Sex: 0.6958  Diet: 0.4288 |
|  | 6dB | 36.127 ±4.132 | 26.558 ±7.081 | 38.740 ±5.702 | 31.934 ±3.259 | Sex*Diet: 0.07255  Sex: 0.6063  Diet: 2.547 | Sex*Diet: 0.7892  Sex: 0.4411  Diet: 0.1190 |
|  | 9dB | 49.875 ±4.977 | 34.224 ±6.155 | 56.566 ±5.947 | 43.902 ±7.999 | Sex*Diet: 0.05267  Sex: 1.582  Diet: 4.733 | Sex*Diet: 0.8197  Sex: 0.2164  Diet: **0.0360*** |
|  | 12dB | 54.513 ±8.126 | 38.714 ±6.471 | 56.443 ±7.112 | 53.185 ±6.442 | Sex*Diet:0.7331  Sex: 1.254  Diet: 1.693 | Sex*Diet:0.3974  Sex: 0.2700  Diet: 0.2013 |
|  | 15dB | 60.375 ±5.521 | 51.279 ±5.484 | 67.085 ±5.309 | 61.394 ±6.866 | Sex*Diet: 0.07966  Sex: 1.944  Diet: 1.502 | Sex*Diet: 0.7793  Sex: 0.1715  Diet: 0.2282 |
